# Enhanced Awareness Followed Reversible Inhibition of Human Visual Cortex: A Combined TMS, MRS and MEG Study

**DOI:** 10.1371/journal.pone.0100350

**Published:** 2014-06-23

**Authors:** Christopher P. G. Allen, Benjamin T. Dunkley, Suresh D. Muthukumaraswamy, Richard Edden, C. John Evans, Petroc Sumner, Krish D. Singh, Christopher D. Chambers

**Affiliations:** 1 Cardiff University Brain Research Imaging Centre, School of Psychology, Cardiff University, Cardiff, Wales, United Kingdom; 2 The Hospital for Sick Children, Toronto, Ontario, Canada; 3 School of Medicine, Johns Hopkins University, Baltimore, Maryland, United States of America; University College of London - Institute of Neurology, United Kingdom

## Abstract

This series of experiments investigated the neural basis of conscious vision in humans using a form of transcranial magnetic stimulation (TMS) known as continuous theta burst stimulation (cTBS). Previous studies have shown that occipital TMS, when time-locked to the onset of visual stimuli, can induce a phenomenon analogous to blindsight in which conscious detection is impaired while the ability to discriminate ‘unseen’ stimuli is preserved above chance. Here we sought to reproduce this phenomenon using offline occipital cTBS, which has been shown to induce an inhibitory cortical aftereffect lasting 45–60 minutes. Contrary to expectations, our first experiment revealed the opposite effect: cTBS enhanced conscious vision relative to a sham control. We then sought to replicate this cTBS-induced potentiation of consciousness in conjunction with magnetoencephalography (MEG) and undertook additional experiments to assess its relationship to visual cortical excitability and levels of the inhibitory neurotransmitter γ-aminobutyric acid (GABA; via magnetic resonance spectroscopy, MRS). Occipital cTBS decreased cortical excitability and increased regional GABA concentration. No significant effects of cTBS on MEG measures were observed, although the results provided weak evidence for potentiation of event related desynchronisation in the β band. Collectively these experiments suggest that, through the suppression of noise, cTBS can increase the signal-to-noise ratio of neural activity underlying conscious vision. We speculate that gating-by-inhibition in the visual cortex may provide a key foundation of consciousness.

## Introduction

### 1.1 Initial behavioural experiment

Blindsight has been one of the most informative conditions in recent investigations of consciousness (e.g.[1], [2], [3], [4]). During blindsight, disruption of early visual cortical areas degrades conscious awareness of stimuli while leaving perception under forced choice conditions relatively preserved. Analysis of the nature of the disruption and the residual capacities has furthered our understanding of the processes that contribute to both conscious and unconscious perception.

Previous demonstrations of transcranial magnetic stimulation (TMS)-induced blindsight have largely involved applying single or short bursts of TMS to the occipital lobe to interfere with subjects' awareness of stimuli (e.g.[5], [6], [7], [8]). This has informed our understanding of the causal temporal dynamics of occipital processing in visual consciousness. Additionally, the demonstration of above-chance perceptual capacity despite such interference is informative with respect to unconscious processing, leading to the suggestion that it may be supported by pathways that bypass the main geniculostriate route.

In contrast to event-related TMS, continuous theta burst stimulation (cTBS) is a repetitive TMS protocol that has been shown to reduce cortical excitability for a more prolonged period (∼45–60 minutes). Previous studies have shown that the cortical response to single TMS pulses is diminished following application of cTBS [9], [10]. Elevations in the principal inhibitory neurotransmitter γ-Aminobutyric acid (GABA) have also been observed following cTBS [11].

The question initially posed by this series of experiments was whether a reduction in occipital cortical excitability, as caused by cTBS, would also impair awareness. Moreover, we sought to discover whether this predicted deficit of awareness would be accompanied by above-chance discrimination of ‘unseen’ stimuli, consistent with previous demonstrations of TMS-induced blindsight.

Contrary to this hypothesis, the initial experiment of this series (Experiment 1) revealed that conscious detection of visual stimuli *increased* following the application of occipital cTBS relative to a sham control condition. ‘Unseen’ discrimination remained above chance but appeared to be unaffected by cTBS. The selective increase in conscious detection as a result of an inhibitory cortical protocol was counterintuitive and the opposite of blindsight, thus warranting replication and deeper exploration. In the following sections we describe the initial experiment and a series of experiments designed to explore and test the replicability of this behavioural effect (Experiments 2–4).

How might a neuronally suppressive protocol increase awareness? Possibly the least informative explanation is that cTBS alters arousal. This could potentially arise as part of a reaction or expectation [12] by subjects to active stimulation, independently of the direct neuronal effects of TMS. Pupil diameter is one of the most commonly used and reliable measures of autonomic arousal [13], [14] and has previously been shown to be modulated by repetitive TMS [15]. If cTBS alters arousal then we would expect see correlated changes in pupil diameter relative to an appropriate control condition.

A more informative interpretation of the behavioural effect might be based upon our current understanding of the effect of cTBS, centred especially on inhibition. It is commonly assumed that increased activity in sensory cortical areas indicates increased conscious representations (e.g. [16], [17]). However, it is possible that relative suppression of sensory representations is also crucial for consciousness. In other words, the dampening or active inhibition of some neuronal processes may bring others into relief and could thus be conducive to optimal detection. If active inhibition is a key determinant in the gating of conscious perception then an inhibitory protocol such as cTBS could plausibly enhance awareness. This hypothesis, denoted hereafter as the *gating-by-inhibition* hypothesis, became the focus of the subsequent experiments in which we also replicated the original effect and explored alternative explanations, such as the possibility that cTBS induced an unexpected *increase* in cortical excitability.

### 1.2 Experiment 2: Assay of cortical excitability

One method used to determine levels of intrinsic neuronal excitability is cortical responsiveness to single pulse TMS [9], [10]. Previously this has been used to demonstrate the effectiveness of cTBS as a suppressive technique in the motor [9] and visual cortices [10]. In the visual domain this involves stimulating the same occipital regions as targeted by cTBS here and then measuring the TMS intensity required to elicit a visual percept known as a phosphene. This procedure results in the calculation of a phosphene threshold (PT) [10]. Franca et al. applied cTBS at 80% of PT, which caused a subsequent elevation of PT [10].

Here we applied cTBS at 80% resting motor threshold [18] to ensure adherence to TMS safety guidelines [19], which prescribe safe intensities of TBS in terms of motor threshold. Since motor thresholds are typically lower than phosphene thresholds [20], [21], the intensity at which cTBS was applied in Experiment 1 was lower than that applied by Franca et al. (here the mean intensity was 40.4% of maximum stimulator output ±5.2SD, whereas Franca et al. applied cTBS at 45.7%±10.9SD). Since reversals of TMS effects, from suppression to facilitation, have been demonstrated when the intensity of TMS is lowered [22], the difference in intensity between our study and Franca et al. raises the possibility that our cTBS protocol may have induced an opposite effect on cortical excitability. In Experiment 2, we therefore attempted to replicate the study of Franca et al. but using the cTBS parameters applied in Experiment 1.

A successful replication of Franca et al.'s observation that cTBS elevates PT would not only confirm the inhibitory after-effect of our protocol but would also be consistent with the gating-by-inhibition hypothesis. In contrast, a reduction in PT would support the idea that the cTBS applied in Experiment 1 added noise to the visual system, resulting in an increased likelihood of any particular representation crossing a threshold for detection. This explanation is known as a ‘stochastic resonance’ effect and has previously been proposed as a mechanism by which TMS can facilitate processing (see [23]). Alternatively, cTBS could simply increase responsiveness of affected areas, which would include neurons involved in representing task-relevant stimuli (e.g. [16]). These last two explanations would predict increased cortical activity and thus run counter to the gating-by-inhibition hypothesis.

### 1.3 Experiment 3: Magnetic Resonance Spectroscopy

Active inhibition most likely involves the principal inhibitory neurotransmitter GABA [24]. The technique of magnetic resonance spectroscopy (MRS) offers the opportunity to quantify *in vivo* GABA concentration and hence to assess the neurochemical balance between excitation and inhibition (see for review [25]).

Previously, MRS has been used to demonstrate an increase in GABA concentration following the application of cTBS to the motor cortex [11], raising the question of whether such effects are reproducible in non-motor cortical areas. Furthermore, the application of MRS to study functional changes in GABA is a relatively new and unconfirmed approach, the implementation of which can differ widely between laboratories. For example, Stagg et al. [11] calibrated their quantification of GABA according to *in situ* N-acetylaspartate (NAA) levels, whereas many previous MRS studies normalise as standard to water concentration [25]. Therefore, the reliability and reproducibility of MRS is central to the development and applicability of the technique as a whole. Elevated GABA concentration following cTBS would lend weight to the gating-by-inhibition hypothesis, whereas reduced GABA would instead suggest increased excitability and the possibility of differential effects of cTBS between cortical regions.

### 1.4 Summary: Magnetoencephalography and behavioural replication

The final experiment of this series aimed to replicate the initial behavioural experiment during concurrent magnetoencephalographic (MEG) recording. MEG offers the opportunity to construct a detailed picture of how cTBS might influence neural processing. Experiment 4 involved the development of several dependent measures to reflect different aspects of neuronal activity. The rationale and hypotheses of these measures is described in a specific introduction (Section 5.3).

Henceforth we report the methods and results of Experiments 1 to 3. The description of Experiment 4 consists of the modified behavioural methods and results, followed by a specific introduction to the MEG data and associated methods and results. Finally, all experiments are interpreted in a general discussion.

To anticipate, the behavioural increase in conscious detection replicated and the experiments involving PT (Experiment 2) and MRS (Experiment 3) supported the gating-by-inhibition hypothesis. For the experiment involving MEG (Experiment 4) we developed several measures for tracking activity profiles that contrast conscious and non-conscious states. We then applied these measures to the contrast involving cTBS, yielding results that were inconclusive but with trends that were consistent with the gating-by-inhibition hypothesis.

## Experiment 1: Behavioural Experiment

### 2.1. Experiment 1: Behavioural Methods

This experiment sought to test the effects of occipital cTBS within a behavioural paradigm capable of revealing TMS-induced blindsight. The experiment included quantification of pupil diameter as an assay of autonomic arousal.

#### Subjects and ethical statement

Sixteen neurologically healthy subjects participated in Experiment 1 for monetary compensation (£10 per hour; 5 females; aged 21–35, *M* = 27.4; *SD* = 3.6). All provided written informed consent and were screened for medical contraindications to TMS, including personal or family history of epilepsy [26]. This research was approved by the Ethics Committee at Cardiff University School of Psychology. All subsequent experiments adhered to this approval and all participating subjects completed the consent and screening procedures.

#### Task

**Figure 1:**
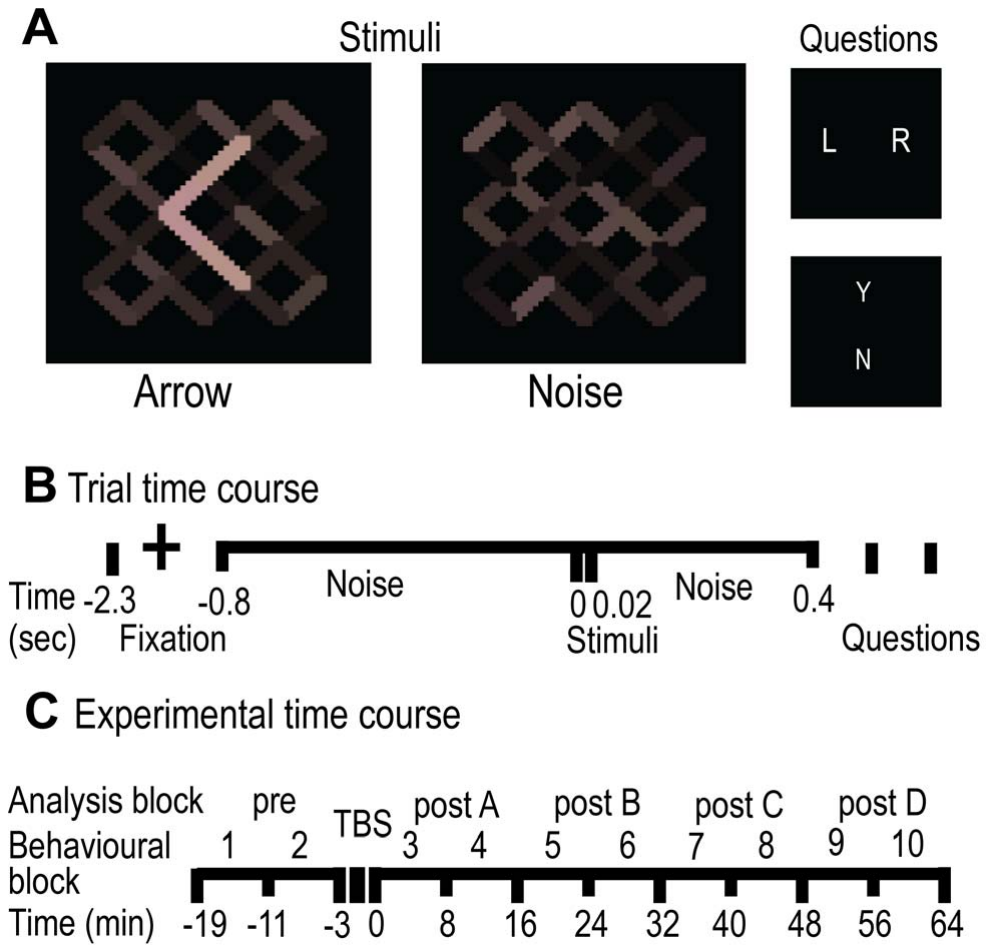
Experimental Design. A. Example of arrow stimuli, noise (stimulus-absent) and task questions. The questions presented on every trial were ‘Was the arrow pointing left or right?’ denoted by ‘L R’ and ‘Did you see the arrow? Yes or No’ denoted by ‘Y N’. B. Time course of each trial. Fixation was followed by noise alternating at 50 Hz with a stimulus frame (20 ms) displayed at 800 ms on half of the trials. Responses to questions followed after a further 400 ms of noise and were not speeded. Questions commenced with the ‘L R?’ decision. C. Time course of the experiment. Behavioural (and MEG acquisition, see Experiment 4) blocks of eight minutes were collapsed into sixteen-minute analysis blocks, to align with the acquisition of MRS (see Experiment 3) and phosphene threshold data (see Experiment 2) acquisitions. Pre-TBS blocks were used to baseline the data. Active and control TMS were applied in separate sessions.

The behavioural task involved presenting subjects with arrow stimuli (Figure 1A) and asking them, (a) to discriminate the direction of the arrow, and (b) if they were consciously aware of having seen the arrow. This composite task allowed us to derive a measure of each subject's conscious awareness of the arrow and a measure of their residual perceptual capacity when reporting not having ‘seen’ the arrow. Insofar as blindsight may be understood as a dissociation between these measures [27], [28], independent fluctuations in conscious detection and ‘unseen’ discrimination following cTBS would have been capable of demonstrating blindsight.

In Experiment 1, visual stimuli were presented using a Cambridge Research Systems Visage and Real Time Sequencer system on a Matlab platform, via a Mitsubishi Diamond Pro 2070sb monitor, refreshing at 100 Hz, which was degaussed and regularly gamma-corrected. The arrow target stimulus was a 20 ms increase in luminance amongst luminance noise (Figure 1B). The noise started 800 ms prior to the target and continued for 400 ms afterwards against a black background, alternating every 20 ms within a range of 17.5 to 32.5 cd/m^2^. The noise also contained a small coloured increment to allow for a potential s-cone manipulation not implemented here [8]. Left and right arrows were presented singularly and in equal proportions, with equivalent luminance at the fixation point subtending the vertical meridian. The noise occupied 1.97°×1.97° visual angle, and the target arrow subtended 0.90°×1.34°. On half the trials (stimulus-absent condition) a noise frame was displayed in place of the target stimulus. After every trial, subjects were first asked in which direction the arrow was pointing (Left or Right?) and then whether or not they were consciously aware of having seen the arrow (Yes or No?) (Figure 1A). Questions were posed in that order to reduce the impact of the latter question upon the former. Responses were recorded via key press on a standard keyboard.

#### Measures

From the questions described above, two principal measures were derived. *PrC* represented conscious awareness of the arrow, calculated through the application of non-parametric Signal Detection Theory (SDT), based on responses to the Yes/No question [29]. We applied SDT because a major criticism of blindsight-type phenomena is that they may represent differences in subjects' response criteria rather than a specific dissociation involving consciousness (e.g. [30], [31]). This concern can be ameliorated if criteria differences are removed from the central measures and analysed independently [29], [32], [33]. Here we used non-parametric SDT due to imbalances in the trial numbers per stimulus condition (see Section 5.1) and response profiles, which violate the assumptions of classic SDT [29].

The application of SDT was as follows: acknowledged awareness in the presence of an arrow was a ‘hit’; acknowledged awareness when no arrow was presented was a ‘false alarm’; denial of awareness when no arrow was presented was a ‘correct rejection’; and denial of awareness when an arrow was present was a ‘miss’. These response categorisations where then used to calculate a hit rate (hit rate  =  hits/stimuli present trials) and a false alarm rate (false alarm rate  =  false alarms/stimuli absent trials). The PrC measure was calculated as hit rate minus false alarm rate [29]. The second principle measure was ‘unseen’ discrimination ability, *PcU*, calculated as proportion of correct judgements of arrow direction when subjects reported not having seen the arrow.

#### Procedure

All subjects completed an initial threshold session during which stimuli and TMS levels were calibrated; this was followed by two experimental sessions on separate days when either cTBS or control (sham) stimulation was applied. The order of the experimental sessions was counterbalanced across subjects.

Behavioural thresholds were set by titrating the luminance of the target arrow so that subjects consciously detected the target at PrC  = 0.6. Following a period of familiarisation with the task and stimuli, the luminance of the target was adjusted in 20-trial runs of 80-trial blocks and repeated for approximately 15 blocks. This produced a psychophysical function to which linear or sigmoidal regression was applied (depending on goodness of fit), which was then solved for the threshold value. A block of 80 trials at the derived luminance value was then completed to confirm the threshold values, and small adjustments were made as required if performance exceeded a tolerance of ±0.15 Pr units. Where adjustments were necessary, blocks were repeated at the new luminance value.

During the main experimental sessions, blocks consisted of 80 trials in a randomised order. Ten blocks were undertaken in each session, including two blocks before and eight blocks after the application of cTBS. Each block lasted eight minutes, including a short rest period. The first block started 1 minute 20 seconds after cTBS had ended, such that data were collected for 65 minutes after the cTBS had finished (see Figure 1C). For experimental sessions, if the performance during baseline pre-cTBS blocks exceeded the ±0.15 PrC tolerance, similar small adjustments were made to the luminance of the stimuli and the pre-cTBS block repeated to maintain performance. If performance exceeded the criteria after four blocks then the experimental session was abandoned in order to avoid additional fatigue effects, and the session rescheduled.

Consistent with previous studies of TMS-induced blindsight [5], [6], [7], [8], a round coil was used to administer cTBS (Magstim High Power 90 mm Coil and Magstim biphasic Rapid^2^ stimulator), delivering 600 pulses over 40 seconds at an intensity of 80% of individual resting motor threshold (following [9]). This corresponded to a mean TMS intensity of 40.4% stimulator output (±5.2% SD). Motor threshold was established using the observation of movement method and was calculated as the average across left and right hemispheres [18]. Coil positioning was achieved using a miniBIRD system (Ascension Technology Corp) in conjunction with MRIcro, MRIreg software and structural MRI scans [34]. These T1-anatomical scans, used in all subsequent coil targeting, were collected on a separate session using a HDx 3 Tesla General Electric MRI scanner (1 mm^3^ isotropic, field of view 256×192×176, TR/TE 7.9/3.0 ms, TI 450 ms, Flip angle 20^o^). Stimulation was targeted at the striate cortex (V1); that is, the closest scalp coordinate to the mid-hemispheric termination of the left and right calcarine sulci. Because of the relatively diffuse effect of a round coil and consistent with previous work [35], the anatomical distribution of the induced current can be confidently attributed only to the occipital cortex generally rather than V1 specifically. In the active cTBS condition, the rim of the coil was positioned 2 cm below the closest scalp coordinate to V1, which centred the rim over V1, with the handle pointing upward and side ‘B’ facing away from the subject. The position was closely matched in the sham (control) condition except that the coil was oriented horizontally and a 10.6 mm spacer inserted between scalp and coil to replicate the contact artefact. This protocol was used in all subsequent applications of cTBS.

#### Pupillometry

Pupil diameter was recorded using an infrared eye-tracker (Cambridge Research Systems 250 Hz chin rest mounted eye-tracker). Pupil diameter was quantified for each behavioural block by averaging the data collected on each trial into a block average, following the filtering for any loss of pupil signal. Eye-tracking also allowed trials in which the subject blinked during the stimuli presentation to be removed from the analysis (identified by a vertical shift in signal followed by a transitory loss of signal coincident with the stimuli presentation). Across all subjects, this criterion resulted in the exclusion of 72 trials from a possible 25,600, which made no appreciable difference to the data and analysis. Two subjects were excluded from this analysis of eye-tracking data owing to failure of the eye-tracker to record reliable pupillometry data.

#### Statistics

Behavioural blocks of 8 minutes were concatenated into blocks of 16 minutes to improve the power/reliability of individual data points and to align the behavioural experiment with imaging acquisitions in subsequent experiments (see Sections 3–5). Effects of cTBS were assessed using repeated measures ANOVAs for each measure in the analysis. The dependent variable in each analysis was the measure of interest (PrC or PcU) for each post-cTBS block, baselined to pre-cTBS levels, thus taking into account day-to-day differences in performance. The factors for the ANOVA were TMS site (2 levels, active *vs*. control) and time from start of the TMS (4 levels, 2–18, 18–34, 34–50, 50–66 minutes). Greenhouse-Geisser corrected *p* values are reported. ‘Unseen’ performance was compared to chance using single sample *t*-tests applied to each analysis block and appropriate Holm-Bonferroni corrections were applied for the multiple blocks/comparisons [36].

In addition to frequentist Neyman-Pearson analyses, the effects of cTBS were assessed concurrently using Bayesian hypothesis testing. Bayesian statistics complement conventional analyses by indicating the confidence that can be placed in both a hypothesis given the data and, crucially, the null hypothesis [37]. The output of Bayesian hypothesis testing is a Bayes factor (B), which, when greater than 3 indicates that the data provides substantial support for the hypothesis and when below 1/3 indicates that the data supports the null [38], [39].

Here, the use of Bayesian hypothesis testing required quantifying the effect of cTBS by constructing a vector that included active cTBS minus control (sham) cTBS, averaged across post-TBS blocks and further subtracted from the pre-TBS baseline. The mean and variance across subjects could then be integrated with *a priori* hypotheses to address the confidence that can be placed in the hypothesis and the respective null. The hypotheses here are represented by uniform distributions ranging from 0 to −0.5 for both PrC and PcU measures [8], [37], representing potential reductions in these measures following cTBS. In this way, cTBS-induced blindsight should be expressed by the Bayes factors supporting the hypothesised drop in PrC, while concurrent PcU should remain unaffected with the corresponding Bayes factor supporting the null. Additionally, the inverse analysis was applied (with priors of range 0 to +0.5) to investigate potential increases in performance. Since the dependent measures were calculated relative to the sham and pre-cTBS baselines, an origin of 0 was selected for the prior distributions. The limit of 0.5 was selected because it represents the maximum reasonable shift in either of the measures: proportion correct of ‘unseen’ discrimination (PcU) cannot exceed 1 and should not be expected to drop below chance (0.5). The measure of conscious detection (PrC) was calibrated to 0.6 and while complete elimination of detection at a PrC of 0 is possible, it was considered improbable based on existing studies [8]. A predicted reduction of PrC to ∼0.1 was judged as a more reasonable limit and aligned the analysis of PrC with that applied to PcU. It is theoretically possible for fluctuations in the dependent measures to exceed these limits, for instance through ceiling-level performance dropping below chance. However, because the measures are twice subtracted from controls (pre-TBS and sham; resulting in the addition of statistical noise upon each subtraction) the expectation that any consistent effect should approach the upper limits of the hypothesised effects was limited. This means that this application of Bayesian hypothesis testing is conservative and should not unfairly favour H1 or H0. Adjustments were made to group level standard errors, as recommended by Dienes [37]. This adjustment was applied to all subsequent applications of Bayesian hypothesis testing. Reported analyses therefore comprise frequentist statistics (*t* or *F*), and where comparisons are between two conditions (e.g. active *vs*. control) the Bayes factors (B) and effect sizes are reported. The effect size here is represented by Cohen's d (*d*), where the variances are pooled across conditions [40], [41].

Outliers were identified and excluded on the basis of Chauvenet's criterion [42], applied to the dependent variable capable of representing a TMS dependent effect, i.e. the same vector to which the Bayesian statistics were applied: [mean of post active cTBS minus pre active cTBS] minus [mean of post sham cTBS minus pre sham cTBS]. This resulted in data representing the distribution in effect sizes following cTBS. To satisfy Chauvenet's criterion, if the probability of any subject's data within this group vector multiplied by the number of samples in that group was less than 0.5 then the subject's data was excluded from the analysis of that measure. All subsequent outlier rejection applied this method and resulting exclusions are reported in the results sections of the corresponding experiments. As reported below, two of the authors (CPGA, CDC) participated in this initial experiment. The exclusion of their data from the analysis did not appreciably affect the outcomes.

#### Statistics for Pupillometry

Frequentist statistics applied to the pupil diameter data followed that applied to the behavioural data. A repeated-measures ANOVA, of the same structure as described above, probed the effects of cTBS. The main hypothesis with respect to the pupillometry data was that an effect on arousal would be expressed by a significant site effect (active cTBS *vs*. control).

The Bayesian analysis was applied to the pupillometry data in three ways, which differ from that applied to the behavioural data. The first of these methods was not only applied to the pupillometry data, but was also used to assess all subsequent dependent measures. Since the change in conscious detection in the behavioural experiment is the effect of central interest in these investigations, the prior used to assess these other dependent measures is based on standardised shift in conscious detection, unless otherwise stated.

This primary Bayesian analysis involved constructing a vector which summarised the cTBS-dependent effect for the current dependent measure, in this case pupil diameter, and the behavioural change as follows: [mean of post active cTBS minus pre active cTBS] minus [mean of post sham cTBS minus pre sham cTBS]. These vectors were then converted to *z* scores, allowing effects and variances to be compared. Because these measures are calculated relative to a sham and pre-cTBS baseline, 0 was the starting point for the theoretical distributions. Moreover, because smaller effects were considered more likely than larger ones, a half-normal distribution was used. Following Dienes [43] the standard deviation of this prior distribution was set as the standardised mean difference of the effect upon conscious detection. As the effect upon the measure of conscious detection was relatively large (0.69 standard deviations), its use represents a conservative (favouring the null) application of Bayesian hypothesis testing, which importantly can be transposed to test effects upon other dependent measures in these experiments. The quantification of the behavioural effect made use of all available data, i.e. concatenating the data across Experiment 1 with a subsequent replication (Experiment 4; described in Section 5.2). In cases where a subject participated in both experimental sections (original and replication; 4 subjects) averages of behavioural performance across experiments were used. Reported together with corresponding *F* statistics are Bayes factors, which represent the confidence that should be placed in the experimental hypothesis (half normal, starting at zero, with variance equal to the behavioural effect upon PrC) over that of the corresponding null [43]. Additionally, the directionality of the hypothesis (determined by the dependent measure in question) is reported (either active>sham or active<sham).

The second method of applying Bayesian statistics was to compare the standardised effects observed in Experiment 1 *only* with standardised fluctuations in pupil diameter. This arguably allows for a more precisely informed prior than when using all behavioural data (Experiment 1 and Experiment 4) because there is a direct correspondence between the behavioural data used in the construction of the prior and the data to be assessed. This method can only be applied to pupillometry data and the MEG data (see Section 5.4) where there is a match between behavioural and other dependent measures.

The final way in which the Bayesian method was applied was to test for an effect relative to an externally defined hypothesis: It has previously been shown that pupil diameter fluctuates in response to changing levels of luminance by as much as approximately 30% of its original diameter [14]. This therefore seemed a reasonable limit for the externally-defined uniform prior representing change in pupil diameter following the application of cTBS [37].

As noted, the behavioural effect of cTBS shown in Experiment 1 was an increase in detection performance. Therefore the informative effect with respect to pupil diameter would be an increase, reflecting a possible increase in arousal. For this reason the application of these Bayesian methods implemented positive priors, corresponding to increased pupil diameter.

#### Summary

The primary hypothesis of Experiment 1 was that occipital cTBS, being a cortically suppressive intervention, should cause an effect resembling blindsight that includes two concurrent behavioural profiles: a reduction in ΔPrC (post-cTBS minus pre-cTBS) following active cTBS compared to sham, together with residual ‘unseen’ capacity (PcU) that is greater than chance and unaffected by cTBS. The interaction of these effects with time following cTBS was of secondary interest. Data for all experiments can be downloaded from http://dx.doi.org/10.6084/m9.figshare.909359.

### 2.2. Experiment 1: Results

**Figure 2:**
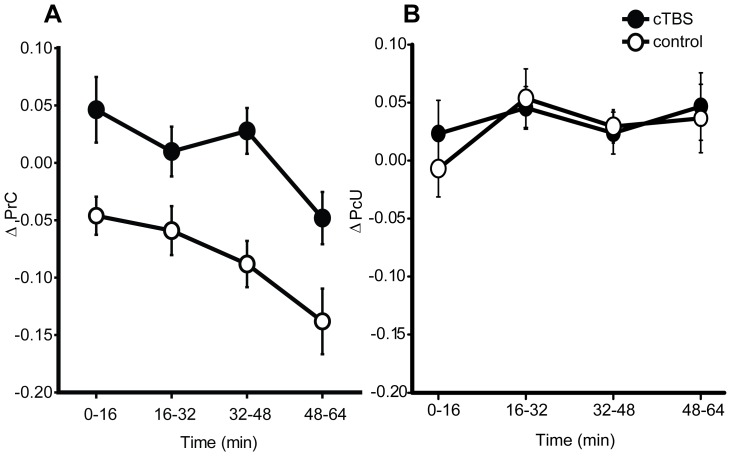
Experiment 1 – psychophysical results. Effects of cortical stimulation on A) conscious detection (ΔPrC; cTBS *vs*. control *p* = 0.002, B_(cTBS>sham)_ = 49.36) proportion correct in reportedly ‘unseen’ discrimination (ΔPcU; cTBS *vs*. control *p* = 0.84, B_(cTBS>sham)_ = 0.08). Data were baselined using pre-TBS performance and illustrate group mean performance following active (cTBS) and control (sham) conditions. Time corresponds to the four trial blocks collected after the TBS was applied. Error bars are ±1 within-subject standard error [122], [123]. All subsequent line plots conform to this structure and are accompanied by corresponding statistics.

Contrary to the blindsight hypothesis, conscious detection (ΔPrC) of stimuli *increased* following application of active *vs*. sham cTBS (*F*_(1,14)_ = 14.02, *p* = 0.002, *d*  = 1.01; see Figure 2; N = 1 outlier excluded). ‘Unseen’ discrimination, although consistently above chance (all *t*_(15)_>7.17, *p*<0.001, relative to Holm-Bonferroni corrected α = 0.005) was not significantly influenced by cTBS (main effect of site: (cTBS *vs*. sham) *F*_(1,14)_ = 0.04, *p* = 0.843, *d* = 0.08, N = 1 outlier excluded). The specificity of this effect for the measure of conscious detection, in contrast to above chance ‘unseen’ discrimination ability, places it in the same class of phenomena as TMS-induced blindsight (e.g. [5]) but in the opposite direction: subjects became *more* aware of the arrow stimuli following a neuronally suppressive intervention.

Bayesian analyses confirmed that the results did not support a drop in conscious detection (B_(PrC cTBS<sham)_ = 0.01), however increased detection was strongly evident (B_(PrC cTBS>sham)_ = 49.36). The analysis of ‘unseen’ discrimination strongly supported the null hypothesis that cTBS did not modulate performance: B_(PcU cTBS>sham)_ = 0.08, B_(PcU cTBS<sham)_ = 0.10.

The exclusion of data acquired from the two authors did not appreciably alter the significant effect of cTBS on conscious detection (ΔPrC for cTBS *vs*. sham *F*_(1,12)_ = 11.55, *p* = 0.005, *d* = 1.00, B_(PrC cTBS>sham)_ = 15.81) or the absence of an effect of cTBS on ‘unseen’ discrimination (ΔPcU for cTBS *vs*. sham *F*_(1,12)_ = 0.02, *p* = 0.90, *d* = 0.05, B_(PcU cTBS>sham)_ = 0.08).

Over the course of the experiment, subjects' conscious detection of the stimuli decreased independently of TMS conditions (time effect *F*_(3,42)_ = 6.51, *p* = 0.002, Figure 2). As this effect did not interact significantly with the TMS condition (site × time interaction *F*_(3,42)_ = 0.37, *p* = 0.78) it is explained most readily by fatigue. This is consistent with reports made by subjects following their participation in this relatively long and demanding experiment. No significant time-dependent effects were observed upon the measure of ‘unseen’ discrimination (time effect *F*_(3,42)_ = 1.74, *p* = 0.20, site × time interaction *F*_(3,42)_ = 0.35, *p* = 0.73).

**Figure 3:**
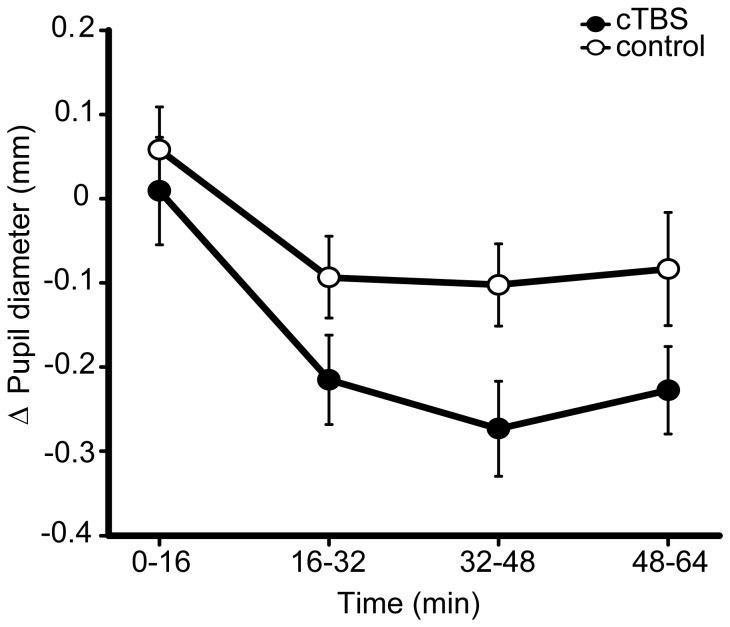
Experiment 1 – pupillometry results. Change in pupil diameter from the pre-TBS baseline, following cTBS and control conditions, over the course of the experiment. cTBS *vs*. control *p* = 0.17, B_(cTBS>sham)_ = 0.231, where prior is based upon behavioural effect across experiments (this prior is used in all subsequent Bayesian statistics described in figure legends, unless otherwise stated). Error bars are ±1 SEM.

The pupillometry data did not support an explanation of the increase in conscious detection based on elevated arousal. Although there was a trend towards a dissociation in the measure of pupil diameter between TMS conditions (site effect (cTBS *vs*. sham) *F*_(1,12)_ = 2.11, *p* = 0.17, *d* = 0.45, N = 1 outlier excluded), the tendency was for a *reduction* following cTBS relative to sham (see Figure 3). Since increased pupil diameter is taken to indicate increased arousal, the direction of this trend suggests that the cTBS-induced increase in conscious detection is unlikely to have stemmed from increased arousal. A similar temporal profile in pupillometry was observed for both TMS conditions: pupil diameter increased following the application of control and active cTBS and then subsided throughout the course of the experiment, indicating its sensitivity to changes in arousal (*F*_(3,39)_ = 9.72, *p*<0.001). This change did not appear to interact with the TMS (site × time interaction *F*_(3,39)_ = 1.02, *p* = 0.37). This reduction in pupil diameter over the course of the experiment, independent of TMS effects, supports the fatigue-based explanation of the time-dependent changes in behaviour, described above.

Corresponding Bayesian analyses revealed no evidence for an increase in pupil diameter following cTBS. Using all conscious detection data to inform the prior (including Experiment 1 and the later Experiment 4), the null hypothesis was strongly supported: B_(Pupil diameter cTBS>sham)_ = 0.23. When the prior drew only upon the behavioural data from Experiment 1, the absence of an effect was supported to an even greater extent: B_(Pupil diameter cTBS>sham)_ = 0.18. Consistent with this negative finding, the externally defined hypothesis of the change in pupil diameter lying between 0 and 30% of its original size also supported the null: B_(Pupil diameter cTBS>sham)_ = 0.06.

## Experiment 2: Phosphene Threshold

### 3.1. Experiment 2: Methods

This experiment sought to replicate Franca et al. [10]. A successful replication would constitute a significant elevation of PT following active *vs*. sham cTBS, confirming that our cTBS protocol induced cortical suppression and providing evidence consistent with the gating-by-inhibition hypothesis. Alternatively, a failure to replicate would indicate a physiological discrepancy between the cTBS applied here and that applied previously, and would further suggest that *increased* cortical excitability may explain the observed enhancement of awareness in Experiment 1.

Twelve subjects participated in the phosphene threshold experiment (aged 19–40, 7 females *M* = 25.3, *SD* = 6.0), three of whom also participated in at least one other experiment.

The intensity of a single TMS pulse required to elicit a phosphene depends on the levels of intrinsic cortical excitability within that region. The method used here to determine PT resembled that of Franca et al. [10]. First, we assessed subjects' susceptibility to phosphenes within safety limits (160% of motor threshold [MT, see 18]). The coil was initially positioned using the miniBird system and tripod as in the behavioural experiment, with single pulses applied at 120% MT. If stimulation did not elicit phosphenes that the subject reported as being ‘reasonably clear’ the coil was moved until it did so, while minimising the distance to the original coil position. This location was recorded using a Brainsight system (Rogue Research Inc.) based on the subjects' anatomical MRI scans. An approximate PT was obtained using an up-down staircase method, applying single pulses approximately every 5 seconds, starting at 50% of maximum output and adjusting TMS intensity in steps of 5%, then 2%, then 1%, so that subjects verbally reported seeing 5 phosphenes from 10 pulses. This level was then used as the starting point for a more thorough threshold estimation procedure where the number of reported phosphenes arising out of 10 pulses was recorded at −10, −5, 0, +5, +10 and +15% of the estimated PT. The orders of these sets of 10 pulses were randomised and the full range of intensities was repeated three times in separate runs separated by short breaks. The coil was repositioned at the start of each run. Averaging across runs yielded a function representing the number of reported phosphenes out of 10 over a range of intensities, to which a regression was applied (sigmoid or linear depending on goodness of fit). Solving this regression for 5/10 phosphenes thus provided the PT.

These sets of three runs comprised a block of data, collected over 16 minutes; this timing was chosen to correspond to the timing of the MRS acquisition in Experiment 3. One block was acquired before the cTBS and three were acquired afterwards. Three rather than four post-cTBS blocks were completed because, even with three post cTBS blocks, the quantity of TMS applied approached the limit permitted by the local ethics committee (based on [19], [26], [44]). Again, sham and active cTBS were applied on separate days, the order of which was counterbalanced across subjects. The mean intensity at which the cTBS was applied for this group was 42.4%±7.4SD.

Inferential analyses were as previously described when applied to the pupillometry data with the following exception. The repeated measures ANOVA included three levels in the time factor, corresponding to there being three, rather than four, post-TBS blocks. Bayesian statistics are as described for the first analysis applied to the pupillometry data; that is, using a prior based upon a standardised behavioural PrC change observed following cTBS across behavioural replications (Experiment 1 and Experiment 4). In addition, we undertook a Bayesian analysis using the effect size obtained by Franca et al. [10] for the prior. Franca et al. [10] reported a change in PT from pre-TBS to post-TBS of +10.6%. The pooled variance of this change can be derived from the pre and post standard deviations as 14.21%. This effect (mean 10.6%±14.21SD) can then be adopted as a Bayesian prior to which changes observed here can be integrated. To implement this analysis, the percent change from pre to post (mean across PTs acquired post-cTBS) was thus calculated (mean 7.89%±11.69SD).

### 3.2. Experiment 2: Results

**Figure 4:**
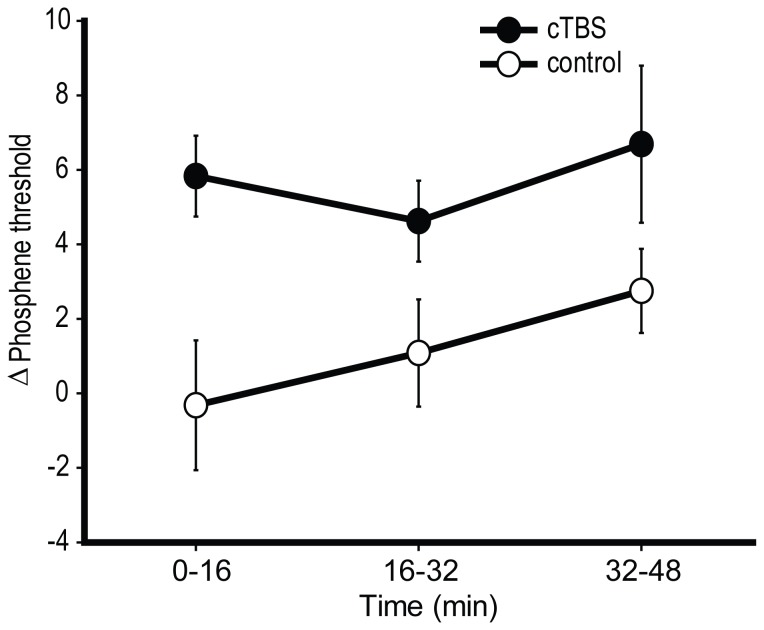
Results of Experiment 2. The change in phosphene threshold from pre-TBS levels, following occipital cTBS and control stimulation. cTBS *vs*. control *p* = 0.04, B_(cTBS>sham)_ = 4.47. Error bars are ±1 SEM.

The TMS intensity required to elicit phosphenes increased significantly following the application of active *vs*. sham cTBS (*F*_(1,11)_ = 5.40, *p* = 0.04, *d* = 0.64, B_(PT cTBS>sham)_ = 4.47; see Figure 4). No significant time-dependent effects were observed (site × time interaction: *F*_(2,22)_ = 1.59, *p* = 0.23, time effect: *F*_(2,22)_ = 0.79, *p* = 0.41). The Bayesian analysis of the replication comparing the changes observed by Franca et al., [10] to those observed here supported the replication, but not unequivocally (B = 2.11).

These results are consistent with the expected inhibitory effect of cTBS, replicating previous observations of Franca et al. [10] but with a marginally reduced magnitude (10.6% reported by Franca et al. compared to 7.9% here). An explanation of the cTBS-induced enhancement of awareness in Experiment 1 according to *increased* excitability or stochastic resonance is inconsistent with these results. Instead, by confirming that cTBS had an inhibitory effect, the results of Experiment 1 are consistent with the inhibition-by-gating hypothesis. The relationship between increased PT and decreased threshold for conscious detection (i.e. the relationship between experiments) is considered in the General Discussion.

## Experiment 3: Magnetic Resonance Spectroscopy

### 4.1. Experiment 3: Methods

This experiment sought to test levels of GABA concentration in the region affected by cTBS. An increase in GABA concentration, relative to control stimulation, would be consistent with increased inhibition and therefore the gating-by-inhibition hypothesis. Additionally it would constitute an indirect replication of previous research [11]. Reduced GABA would instead suggest increased cortical excitability in opposition to the gating-by-inhibition hypothesis.

Data was acquired from 18 subjects (aged 20–40; 7 females; *M* = 26.3, *SD* = 5.0), of whom 12 participated in at least one other experiment. Since Stagg et al. reported a significant effect of cTBS on GABA concentration with N = 8, we chose our sample size on the basis of expecting a replication to require at least twice as many subjects [45].

MRS data was acquired on the 3T GE MRI scanner over two separate sessions (cTBS and sham control), with the session order counterbalanced across subjects. As in Experiment 1, cTBS was administered at 80% adjusted MT, at a mean intensity of 40.8%±5.0SD maximum stimulator output. Unless otherwise stated, the TMS apparatus and cTBS protocol were identical to Experiment 1.

Each session included four MRS acquisitions, with the first obtained prior to cTBS to provide a within-subject, within-session baseline. The cTBS was performed in the MRI control room and the subject was then immediately transferred to the scanner. MR localisation and calibration scans commenced 2 minutes 40 seconds after the start of the cTBS, and the first MRS acquisition commenced 1 minute later (i.e. 3 minutes 40 seconds after the start of the TBS). During the MRS acquisitions all subjects watched the same film and did not perform any behavioural task.

**Figure 5:**
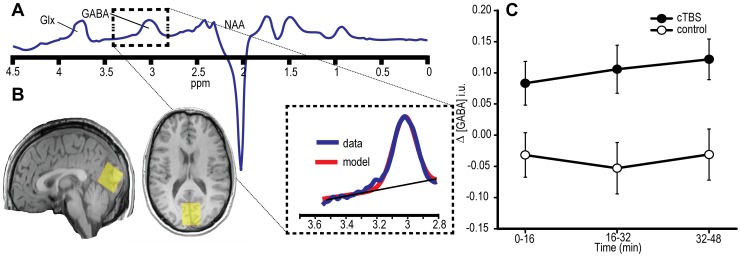
Results of Experiment 3. A) Illustration of model fitting applied to MEGA-PRESS edited spectra that allowed for quantification of GABA concentration. Units are parts per million (ppm) of proton frequency. Glx is the combined glutamate and glutamine peak. NAA is the peak caused by N-acetyl aspartate. B) Illustration of the typical MRS voxel placement used here, as shown in the sagittal and axial view of one participant. C) Average change in GABA concentration followed occipital stimulation. The ordinate indicates change in GABA in institutional units (i.u) relative to the pre-TBS baseline, plotted according to the TMS condition (active cTBS *vs*. Sham control) and time after stimulation (mins). cTBS *vs*. control *p* = 0.034, B_(cTBS>sham)_ = 5.60. Error bars are ±1 SEM.

Before the pre-cTBS MRS acquisition, a T1-weighted anatomical scan was obtained in each subject (1×1×1 mm^3^ isotropic). This allowed individual positioning of the 3×3×3 cm^3^ MRS voxel over V1, which was defined according to the calcarine sulcus as in Experiment 1. The voxel was positioned to avoid non-brain tissue, and so that the lower edge followed the cortical surface and did not enter the cerebellum (Figure 5B). This voxel position was recorded relative to anatomical landmarks in three dimensions using a screen shot and repeated for subsequent acquisitions. Each MRS acquisition lasted 16 minutes and comprised a MEGA-PRESS [46] sequence with the following parameters; 512 transients echo time  = 68 ms, repetition time  = 1800 ms, acquisition bandwidth  = 5 kHz, 4096 FID points, 2 phase cycles, 16 ms editing pulses alternating at 1.9 and 7.5 ppm to separate the GABA molecule from other chemicals [25]. Eight unsuppressed water transients were acquired at the end of each MRS scan to act as a concentration reference.

GABA concentration was quantified from the MEGA-PRESS edited spectra using Gannet [47] (Figure 5A). This involved fitting a Gaussian distribution with a linear baseline component to the GABA peak at 3 ppm, with the area under the curve, relative to that of water, providing quantification of GABA concentration in institutional units (see [46]). It is worth noting that normalisation to water, while standard practice, differs from the Stagg et al., study which used *in situ* NAA for this purpose [25].

Sessions containing unacceptably poor quality data were repeated (2 sessions, owing to subject movement during acquisition). Data quality was checked (for movement and lipid contamination artefacts) and fit quality was improved by manual phasing of the spectra, where the analyst (CJE) was blinded to the TMS condition. Conventional statistical analyses followed the approach of Experiment 1 and 2, with the effects of cTBS assessed by comparing pre-TBS baselined data between control and active conditions in a repeated measures ANOVA. This ANOVA included post-TBS time as a factor but, as in Experiment 2, the primary hypothesis addressed the contrast of site (active *vs*. sham). The expectation, following Stagg et al. was that cTBS would lead to elevated GABA concentration and a significant site effect. The analytic structure of the Bayesian hypothesis testing matched that of Experiment 2 and the pupillometry data, using the standardised behavioural effect to inform the positive prior, thus representing increased GABA concentration. An additional analysis of our data using a prior based on the effect reported by Stagg et al. would be consistent with the Bayesian approach and the analyses previously implemented. However, we were unable to compute an effect size from Stagg et al. [11], therefore such an analysis could not be implemented here.

### 4.2. Experiment 3: Results

Baseline-corrected GABA concentration in occipital cortex increased significantly following the application of occipital cTBS relative to sham cTBS (site effect: *F*_(1,16)_ = 5.347, *p* = 0.034, *d* = 0.70 B_(GABA cTBS>sham)_ = 5.60; see Figure 5C; N = 1 outlier excluded). No time-dependent effects were observed (site × time interaction: *F*_(2,32)_ = 0.416, *p* = 0.603, time effect: *F*_(2,32)_ = 0.347, *p* = 0.656).

These findings in visual cortex indirectly replicate the increase in GABA concentration in motor cortex reported following cTBS of M1 [11]. Moreover, they are consistent with the gating-by-inhibition hypothesis that an increase in inhibitory processes may underlie the enhancement of conscious detection following occipital cTBS.

## Experiment 4: Behavioural replication and MEG

Next we report the attempted replication of Experiment 1 (5.1–5.2), carried out inside the MEG scanner. The remainder of this section addresses the MEG data specifically. The MEG introduction and methods sections contain descriptions of the development of the dependent variables designed to isolate neural correlates of consciousness (5.3–5.4), which are then applied to the contrasts involving cTBS (5.5).

### 5.1 Experiment 4: Revised Methods for Behavioural Replication

Experiment 4 followed the behavioural methods described in Experiment 1, variations to which are now described. A potential criticism of the method adopted in Experiment 1 is that subjects were clearly aware of the difference between the sham and active cTBS conditions. The increase in conscious detection might therefore have arisen due to a reactive response [12] by the subjects to active TMS. One alternative to sham TMS is the selection of an appropriate control site to replicate the auditory and tactile artefacts of stimulation. However, owing to the geometry of the round coil, the induced activation is broadly distributed, as are the processes involved in consciousness. This approach was therefore discounted in the current experiments: wherever the coil was placed, this relative lack of focality could be sufficient to disrupt perceptual or cognitive processes.

Instead, we used intermittent theta burst stimulation (iTBS) as an alternative active control. During iTBS the same number of pulses are applied at the same intensity as cTBS, but with a temporal profile that includes intermittent (8 second) gaps between continuous bursts of 2 seconds. In the motor cortex the physiological effect of iTBS opposes that of cTBS, reliably increasing cortical excitability [9]. For reasons that are not clear, this potentiating effect does not appear to be reproducible in the occipital cortex [10]. Nevertheless, evidence that iTBS produces either opposite or null effects on cortical excitability makes it an ideal occipital control condition to achieve effective participant blinding.

This additional control condition meant that the analysis of TMS effects could potentially have contained three levels (cTBS, iTBS and sham); however our primary questions concern the efficacy of cTBS, and in particular its effect upon the PrC measure. For this reason, the first analysis to be applied tested the equivalence of the iTBS and sham conditions, as controls, using both the rm-ANOVA and Bayesian statistics (using a uniform prior with a 0 to 0.5 range) applied to the iTBS *vs*. sham contrast involving the central PrC measure. Statistical invariance of the two control conditions (iTBS and sham), as determined by Bayesian testing, would justify their treatment as a single collapsed control condition [48]. Following such a demonstration, all other dependent measures would then be analysed in terms of cTBS *vs*. control, where “control” denotes the mean of the iTBS and sham conditions. If such an analysis indicates differences of interest then subsequent *post hoc* analyses may be applied involving the iTBS and sham conditions separately. The order of the different TBS conditions was counterbalanced across sessions between cTBS, iTBS and sham.

The procedure in Experiment 4 was altered in several ways from Experiment 1. The number of subjects was increased from 16 to 24 (aged 19–32; 13 females; *M* = 25.0, *SD* = 3.9). An additional four subjects enlisted for the experiment but did not complete it either due to mild adverse reactions to TMS involving nausea (one subject) or an inability to maintain performance in the behavioral task (one subject) and/or data quality in that head movement regularly exceeded 5 mm from its initial position within an acquisition block (two subjects). Another adaptation was that subjects were thresholded to a level of PrC  = 0.5 (as opposed to 0.6 in experiment 1) in order to optimise the sensitivity to detect both increases as well as decreases in detection ability. Also, the ratio of stimulus-present to stimulus-absent trials was changed from 50:50 to 60:40 in favour of stimuli-present trials in order to optimise the power for the MEG analysis of the stimulus-evoked electrophysiological response.

Responses were collected via a LUMItouch™ response pad. Owing to the need to change rooms (TMS was administered outside the MEG magnetically shielded room) and the time required to localise the subject's head before recording MEG data on every block, a delay of three minutes occurred between the termination of cTBS and commencement of the behavioural task. Subjects were instructed to maintain the same head position as closely as possible during the experimental session. The mean TMS intensity was 41.9% stimulator output (±5.7 SD).

Experiment 4 made use of a SensoMotoric Instruments infrared 50 Hz eye tracking system mounted on a tripod. The time cost of relocating the subject into the magnetically sealed room following TBS and head localisation procedures prevented eye-tracking calibration; therefore eye tracking data were analysed across blocks rather than within individual trials. For this reason, the units of pupil diameter in Experiment 4 are arbitrary rather than in mm, and trials where the subject blinked during stimuli presentation could not be excluded. The pupillometry data was filtered for losses of signal. Pupil diameter was averaged across blocks and the dependent variable was change in pupil diameter from the pre-TBS baseline.

The analysis approach for both the behavioural and pupillometry replication was the same as Experiment 1. Additionally, following Dienes [43], combined Bayes factors are reported in which Bayes statistics collected in Experiment 1 are multiplied by those in Experiment 4 to provide a cumulative estimate of evidence in support of H1 *vs*. H0.

### 5.2. Experiment 4: Behavioural Results

**Figure 6:**
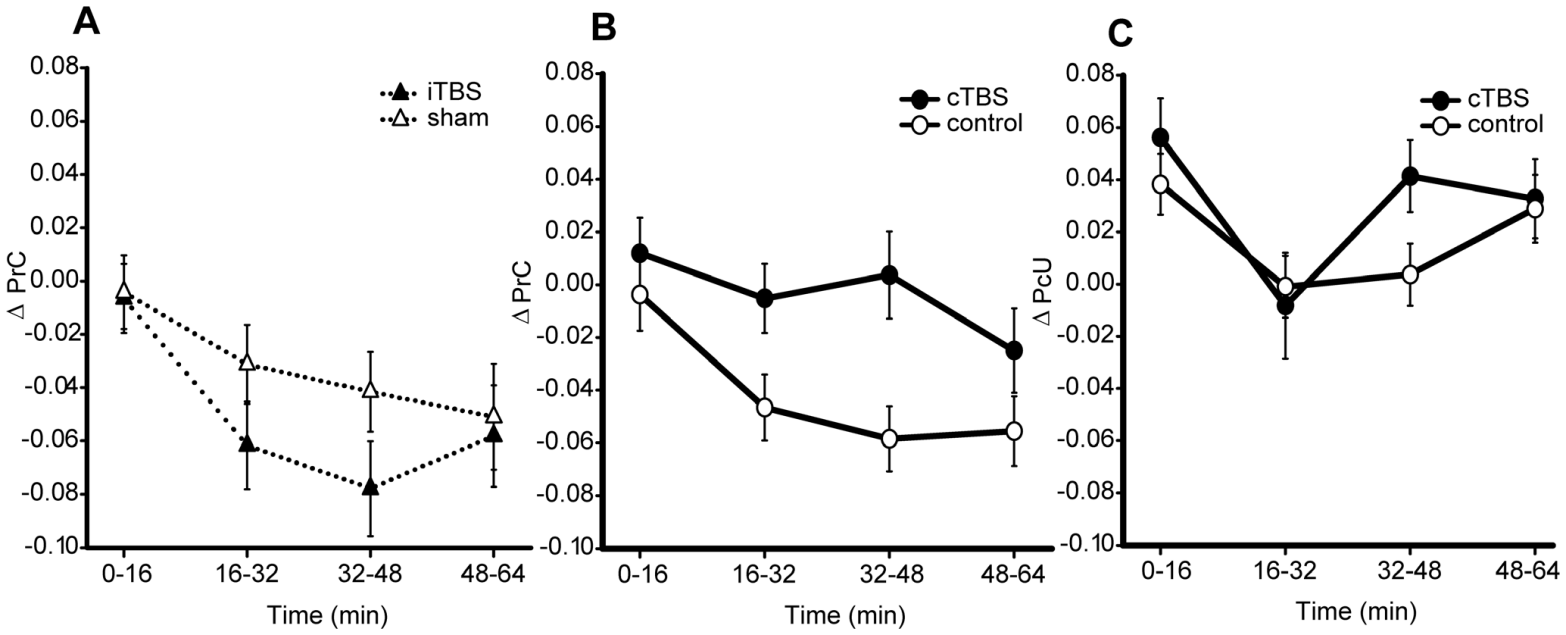
Experiment 4 – psychophysical results. A. Conscious detection (PrC) over the course of the experiment, subtracted from pre-TBS baseline, in the two ‘control’ conditions iTBS and sham (iTBS *vs*. sham *p* = 0.51, B_(iTBS>sham)_ = 0.025, based upon uniform 0–0.5 prior). B. Conscious detection over the course of the experiment, subtracted from the pre-TBS baseline, contrasting cTBS with the collapsed control condition (mean of iTBS and sham). The results replicate the cTBS-induced increase in PrC observed in Experiment 1 (cTBS *vs*. control *p* = 0.031, B_(cTBS>sham)_ = 0.99, based upon uniform 0–0.5 prior). C. Reportedly ‘unseen’ discrimination ability (PcU), subtracted from pre-TBS baseline, under the cTBS and collapsed control condition. cTBS *vs*. control *p* = 0.478, B_(cTBS>sham)_ = 0.09. Error bars are ±1 SEM.

The equivalence of the iTBS and sham conditions was first tested to determine their eligibility to be collapsed into a single control condition. No significant effect of TMS site or interaction with time was observed upon the measure of conscious detection (iTBS *vs*. sham: *F*_(1,22)_ = 1.07, *p* = 0.31, *d* = 0.27, site × time interaction *F_(_*_3,66)_ = 0.72, *p* = 0.51, B_(PrC iTBS>sham)_ = 0.03, B_(PrC iTBS<sham)_ = 0.13, see Figure 6A; N = 1 outlier excluded). Since these analyses, and in particular the Bayesian statistics, provide strong evidence for invariance between these conditions, iTBS and sham conditions were collapsed into a single control condition for subsequent comparison with cTBS.

Behaviourally, both the increase in conscious detection and the apparent absence of an effect upon the measure of ‘unseen’ discrimination replicated (see Figure 6). Conscious detection was higher following the application of cTBS relative to a mean of iTBS and sham (site effect cTBS *vs*. control *F*_(1,23)_ = 5.31, *p* = 0.03, *d* = 0.50; Figure 6B), although the corresponding Bayesian analysis was inconclusive (B_ (PrC, cTBS>control)_ = 0.99). However, taken together with the original experiment the combined Bayes factor (see [43]) (B_(PrC, cTBS>control)_ = 48.97) provides strong evidence for a cTBS-induced increase in conscious detection.

The increase in conscious detection was also apparent when comparing all three TMS protocols (site effect (cTBS, iTBS, sham) *F*_(2,44)_ = 3.61, p = 0.04, N = 1 outlier excluded). This effect appeared to be driven by (individually non-significant) differences between the cTBS and the other conditions (cTBS *vs*. iTBS *F*_(1,23)_ = 4.13, *p* = 0.05, *d* = 0.50, B_(PrC cTBS>iTBS)_ = 0.71, cTBS *vs*. sham *F*_(1,23)_ = 3.40, *p* = 0.08, *d* = 0.40, B_(PrC cTBS>sham)_ = 0.44).

As in Experiment 1, performance was greater than chance when subjects denied awareness of the stimuli under all TMS conditions (*t*_(23)_>8.64, *p*<0.0001 relative to Holm-Bonferroni corrected α = 0.0033). ‘Unseen’ discrimination again appeared to be unaffected by the TMS (site effect cTBS *vs*. control *F*_(1,23)_ = 0.52, *p* = 0.48, *d* = 0.21, B_(PcU cTBS>control)_ = 0.09, site × time interaction *F*_(3,69)_ = 1.13, *p* = 0.34, Figure 6B).

Subjects' conscious detection decreased over the course of the experiment (time effect *F*_(3,69)_ = 3.50, *p* = 0.033; Figure 6A). Since this effect was apparent for all TMS conditions and did not interact significantly with the application of cTBS (site × time interaction *F*_(3,69)_ = 1.36, *p* = 0.26), the most likely explanation of this effect is increasing fatigue, which was consistent with the discursive reports made by the subjects following the experiment. ‘Unseen’ discrimination also appeared to change throughout the course of the experiment (time effect *F*_(3,69)_ = 5.66, *p* = 0.002). This change did not depend significantly upon the application of cTBS (site × time interaction *F*_(3,69)_ = 1.13, *p* = 0.34).

**Figure 7:**
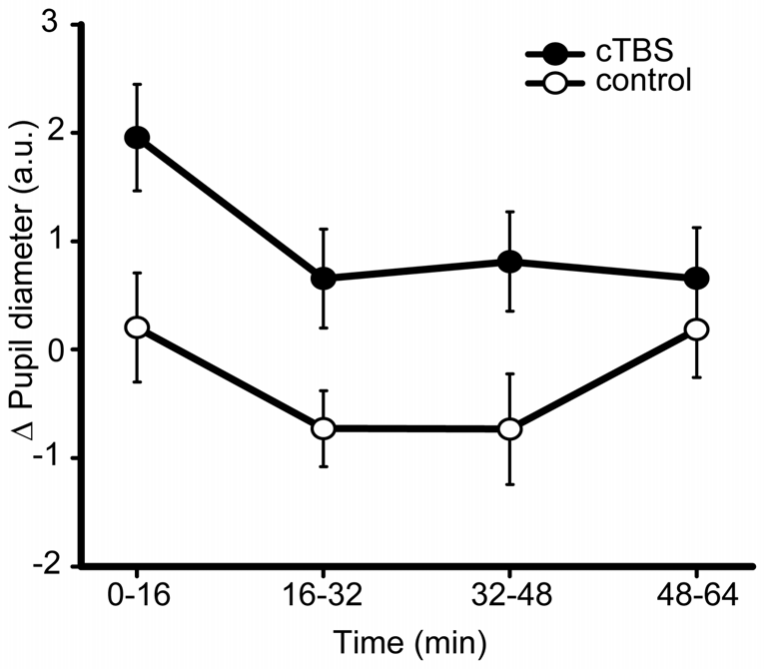
Experiment 4 – pupillometry results. Mean change in pupil diameter from pre-TBS baseline, following cTBS and the collapsed control condition. Units are arbitrary (a.u.). cTBS *vs*. control *p* = 0.21, B_(cTBS>sham)_ = 0.96. Error bars are ±1 SEM.

In contrast to Experiment 1, average pupil diameter in Experiment 4 was greater following cTBS relative to control, although this difference was not statistically significant (site cTBS *vs*. control *F*_(1,22)_ = 1.67, *p* = 0.21, *d* = 0.23, Figure 7, N = 1 outlier excluded). The change in pupil diameter across the session was consistent with Experiment 1 (time effect *F*_(3,66)_ = 7.116, *p* = 0.001), and this change did not interact significantly with the TMS condition (site × time interaction in replication experiment F_(3,66)_ = 1.70, p = 0.20).

Bayesian analyses of pupil diameter that made use of the behavioural effect to derive a prior were inconclusive, both when the combined behavioural set from Experiments 1 and 4 was used to inform the prior (B_(Pupil diameter cTBS>control)_ = 0.964) and when Experiment 4 was considered alone (B_(Pupil diameter cTBS>control)_ = 1.238). However, when the data were integrated with the externally derived prior (where the maximum reasonable shift in pupil diameter was 30%) support was shown for the null (B_(Pupil diameter cTBS>control)_ = 0.223). The combination of Bayes factors across Experiment 1 and Experiment 4 confirmed substantial support for the null hypothesis of no effect of active cTBS upon pupil diameter and therefore arousal levels (using the combined data set to inform the prior: B_(Pupil diameter cTBS>control)_ = 0.223, using the respective behavioural data sets to inform the priors: B_(Pupil diameter cTBS>control)_ = 0.217, using the externally defined 0–30% uniform prior: B_(Pupil diameter cTBS>control)_ = 0.0134). Since pupil diameter was not reliably affected by TMS, the modulation of arousal does not appear to be a viable explanation for the enhancement of conscious detection following occipital cTBS.

### 5.3 Experiment 4. MEG Introduction

Experiment 4 was a replication of Experiment 1, conducted concurrently with MEG to provide a more detailed picture of the neuronal basis of changes in conscious detection. The richness of MEG data provides many potential measures of the effects of cTBS. To constrain these, we developed dependent variables that were optimised to reveal an orthogonal contrast collapsed across TMS conditions: the difference between when subjects reported awareness of a visual stimulus and when they reported not having ‘seen’ the stimulus yet were able to correctly discriminate its identity. This difference mirrors the dissociation of blindsight [27], [28], drawing upon previous research that has used similar techniques to probe the neural correlates of consciousness (NCC [49]). In doing so we aimed to express the maximal difference between conscious perception and perception specifically lacking awareness.

The MEG analysis focused on two features of individual cortical responses to stimuli: the evoked or event-related field (ERF) responses and the induced oscillatory responses. Oscillatory responses were divided into three frequency bands, producing four areas for the MEG analysis: i) later evoked responses corresponding to the ∼M3 component; ii) high frequency event-related synchronisations (ERS) in the γ band from roughly 30–100 Hz; iii) low frequency event related desynchronisation (ERD) in the α band at approximately 6–12 Hz; and iv) low frequency ERD in the β band at approximately 12–30 Hz. The development and motivation for choosing these features are now described.

#### Evoked responses

Of the many NCC's proposed, perhaps some of the most widely acknowledged have involved relatively late (>∼100 ms) cortical electromagnetic evoked responses to stimuli [50], [51], [52]. One of the clearest demonstrations of this relationship is work of Sergent and colleagues using the attentional blink paradigm [53]. They subtracted the electroencephalographic (EEG) trace in the absence of stimuli from those collected in the presence of stimuli, and showed that both reportedly ‘seen’ and ‘unseen’ trials resulted in comparable early (P1 and N1) evoked responses, whereas the later N3/4 (∼300 ms) components were potentiated when the subjects reported the stimuli as ‘seen’. These late evoked responses were thus associated specifically with conscious processing.

In contrast, there is some evidence that earlier potentials are also modulated according to the presence/absence of conscious processing [52], [54], [55], although these studies also demonstrated later awareness-dependent effects. Therefore, there appears to be a broad consensus that beyond ∼200 ms the amplitude of evoked responses reflects the extent to which information is processed consciously [51], [56], [57]. These processes may reflect the passage of conscious information in a recurrent occipital-frontal exchange [51], [55], [58]. Our study was incapable of probing early evoked components as they were not clearly observable across the group. This is likely to be due to the stimuli being presented at peri-threshold levels, combined with their (necessarily) foveal presentation resulting in the cancellation of early evoked responses across the two hemifields [35].

Here we expected the amplitude of the late evoked responses to be greater when stimuli are reportedly ‘seen’. With respect to the TMS effects, a motivation for the quantification of late evoked responses was to test the ‘increased activity’ hypothesis. According to this account, rather than suppressing superfluous representations, occipital cTBS potentiated conscious representations directly, and this may involve up-regulation of recurrent fronto-occipital processing.

#### Oscillatory responses

Oscillations in magnetic activity at the scalp have been associated with a variety of brain processes, dissociable according to frequency. Higher frequencies, such as those in the γ range (∼30–100 Hz) have been linked with the representation and passage of explicit information throughout the brain (e.g. [49]). Lower frequencies in the α and β range, by contrast, have been linked to the active suppression of superfluous information and selection (e.g. [59]), as well as functional connectivity [60]. Therefore, quantification of these induced oscillatory responses may reflect the hypotheses of increased cortical excitability and gating-by-inhibition, respectively.

Perhaps the oscillatory responses most commonly associated with consciousness are in the γ band. This may be due to the proposal that γ frequency oscillations act to convey information between brain areas and bind information into discrete percepts [49], [61], [62], [63]. First person methodologies, in particular, have been used to show correlations between γ band synchronisation and specifically subjective fluctuations in perception [64]. Nevertheless, it is worth noting that similar functions have also been linked with lower frequency oscillations [65] and there may be multiple forms of γ band response with distinct functionality not captured here [66]. An additional motivation for the inclusion of a dependent variable based on γ oscillations is existing evidence of a relationship between γ responses and levels of GABA concentration [67]. More generally, GABA may be central to the governance of neuronal oscillations [68]. This suggests that an increase in GABA concentration following cTBS could potentially be accompanied by γ fluctuations.

Since the magnitude (increased synchronisation) of γ responses is thought to track levels of awareness [69], we expected oscillatory amplitude to be greater when subjects reported awareness of task-relevant visual stimuli. The increase in awareness following cTBS may also be expected to result in an increased γ band response. If γ frequency oscillations are understood as conveyers of conscious signals then such an effect may lend weight to the increased responsiveness hypothesis [70]. However, the association between γ band changes and increased responsiveness should be tempered if γ frequency oscillations act to segment percepts, which may more closely resemble the gating-by-inhibition hypothesis [63], [71]. Furthermore, if levels of neuronal ‘noise’ are suppressed by cTBS (as per the gating-by-inhibition hypothesis) then, theoretically, the capacity of MEG to detect neuronal responses such as those in the γ band may also be amplified. Under either interpretation, potentiation of the γ band response following cTBS would serve as an important mechanistic clue as to the basis of the effects in question.

Historically, low frequency oscillations have been associated with the absence of cortical processing due to their relative dominance during periods of inactivity, or localisation to areas not associated with task performance [72], [73]. This therefore suggested that their role may be one of an ‘idling’ rhythm [74]. The demonstration that low frequency oscillations are causally involved in the determination of whether or not peri-threshold stimuli are perceived and acted upon [75], [76], [77] has changed our understanding of their function, with an emphasis on their active role in the suppression of superfluous information [59] or gating [78]. This role can be seen as reflected in the Event Related Desynchronisation (ERD) [79]. The ERD is a commonly observed phenomenon where there is a shift from a synchronised state – where suppression is imposed – to a relatively desychronised state following the presentation of stimuli. Quantification of these ERDs in both the α and the β bands was the target of dependent variables iii and iv.

The differences in the roles played by α and β oscillations in the visual domain are unclear. In general, β rhythms have been more closely associated with the maintenance of on-going states rather than the absence of processing [80]. This functionally subtle difference does not impact greatly upon the current rationale. Visual ERDs are expressed in both α and β bands and are understood as indicating an active change from a state of suppression. The α and β bands may, however, be isolated through differences in their spatial and temporal distributions (see Section 5.4).

The extent of ERDs has been shown to follow reported awareness of stimuli [81], illusory motion [82] and recovery from comatose states [83]. Furthermore, the ERD response has been shown to dissociate from Event Related Synchronisations (ERS) in these bands, which, by contrast, have been observed following both conscious and unconscious processing [84]. We therefore expected ERDs in both the α and β bands to be of greater magnitude when subjects reported awareness of the stimuli.

Because of the link between low frequency oscillation and active selection or gating, potentiation of ERDs (measures iii and iv) following cTBS would be consistent with the gating-by-inhibition hypothesis. Furthermore, four recent studies have indicated that the application of cTBS to motor areas leads to modulation of β band responses [85], [86], [87], [88], indicating that low frequency responses might also express a cTBS-dependent change here.

The adjustment of dependent variable parameters to fit a desired hypothesis is a problem that especially afflicts psychology and neuroscience [89], [90], [91]. In particular, certain factors such as when or what frequency ranges are selected to contribute to a dependent variable can be manipulated in order to provide evidence in favour of a preferred hypothesis. Here we confront this issue by making the optimisation explicit but doing so according to the orthogonal ‘Seen’ *vs*. ‘Unseen’ correct contrast, independently of the primary question as to the effect of the cTBS. The measures and their parameters were therefore finalised prior to their application to the TMS dependent contrast.

### 5.4 Experiment 4. MEG Methods

MEG was acquired on a 275-channel radial gradiometer system (CTF MEG, MEG International Services Ltd) sampled at 1200 Hz, analysed as 3^rd^ order synthetic gradiometers [92]. At the time of recording, two channels were not working. Data sets were collected in single 8-minute blocks with head localization procedures applied at the beginning and end of each block. Pairs of 8-minute blocks were then concatenated into single analysis blocks, resulting in 16-minute data sets that were consistent with the duration of acquisitions in Experiments 1–3. Trials were epoched from −2.3 to +1 seconds relative to the stimuli onset (see Figure 1) and band-pass filtered with a 1–300 Hz Butterworth filter. This resulted in 15 datasets for each subject (pre, post 1, post 2, post 3, post 4× three TMS conditions of cTBS, iTBS and sham). Data were visually inspected and clearly corrupted data (e.g. from movement) were removed on a trial-by-trial basis. Together with other data loss, this resulted in a mean of 151.6 trials per data set (±12.2SD) equivalent to a 5.2% data loss. These data sets were then concatenated across TMS conditions. From these larger datasets two sub-sets of data were drawn; the first consisted of all trials where the subject reported having ‘seen’ the stimuli and the second was composed of all reportedly ‘unseen’ trials where the direction of the arrow was correctly identified. These data sets were then randomly down-sampled such that for each subject an equal number of trials contributed to both, resulting in two data sets per subject, with each data set containing a mean of 440 trials (±94SD). Statistical analysis was conducted in sensor space and channels were clustered according to their CTF designation. All analyses were applied to the occipital/parietal cluster, unless otherwise stated, as these channels covered the region directly affected by the TMS.

The differences between ‘seen’ and ‘unseen’ correct trials were captured using these data sets; however, in order to avoid selecting independent sources for each condition, data sets combined across ‘seen’ and ‘unseen’ correct trials were produced for the purposes of channel selection. The channel showing the peak evoked or induced responses for each dependent measure in the combined data set was selected, and then passed to the ‘seen’ *vs*. ‘unseen’ correct analysis. A set of dependent measure parameters (frequency band pairings, temporal epochs) were derived based on task restrictions and differences apparent in the data that conformed to previous research (see Oscillatory responses below). Each of the dependent measures with respect to the ‘seen’ *vs*. ‘unseen’ correct contrast are now considered in turn.

#### Evoked response

The data was band pass filtered at 1:40 Hz (3^rd^ order Butterworth). The baseline used was the mean field strength for each channel during the 500 ms prior to the stimulus onset. This baseline period was applied to all MEG dependent measures. Evoked responses for each data set were measured by the peak deflection from baselines applied to data sets averaged across stimulus-present trials. The peak was defined as the maximum amplitude (positive or negative) of the channel in the cluster between 100 ms and 400 ms post stimulus. As the visual noise ended at 400 ms all parameters of the dependent measures were restricted to before this point to avoid conflation with the behaviourally responsive phase of processing and/or the neuronal response to the offset of the stimuli. This temporal restriction constrained the dependent measure to a combination of M3 and M4 components, which have been linked to conscious signal processing [53], [93]. Because this dependent measure made use of no further parameters these constraints were applied to both the channel selection in the combined data set and the ‘seen’ *vs*. ‘unseen’ correct contrast.

**Figure 8:**
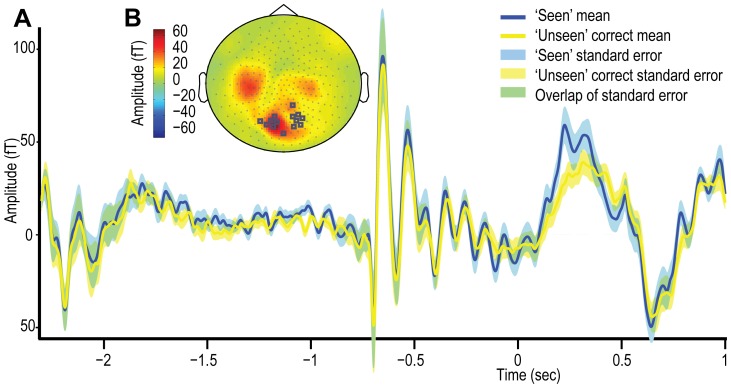
Experiment 4 – ERF results. A. The group averaged event related field (ERF) over the course of ‘seen’ and ‘unseen’ correct trials across TMS conditions. Data was band pass filtered (1 to 40 Hz). Shaded areas corresponded to standard error across subjects. B. Topographic representation of ERF distribution and channel section. The combined (across ‘seen’ and ‘unseen’ correct trials) data was used for channel selection, where the channels which expressed greatest deflection from baseline (−0.5 to 0 sec) in 0.1 to 0.4 second critical period following stimuli presentation were used.

The channels used were selected on the basis of combined evoked responses and are shown in Figure 8B. Figure 8A shows the group averaged evoked response for both ‘seen’ and ‘unseen’ correct trials; clearly apparent is the dissociation between the two traces following the presentation of the stimuli. This difference was quantified by the peak evoked dependent variable which was highly consistent over subjects and statistically significant (*t*_(22)_ = 10.81, *p* = 2.87e^−10^, *d* = 1.29; see Figure 8A). This dependent variable is consistent with previous research (e.g. [51], [56], [57]) and therefore appropriate for application to the TMS contrast.

#### Oscillatory responses

Acquisition parameters are as described above. Time-frequency representations were generated by a stepwise application of the Hilbert transform to generate an analytic function representing the amplitude envelope within specific frequency ranges. To probe γ responses, this procedure used an 8 Hz bandwidth and 2 Hz step size applied to data that was down sampled to 300 Hz. For α and β frequencies, the bandwidth was also 8 Hz but the step size was 0.5 Hz and the data down-sampled to 600 Hz. From each channel and frequency band pair, a baseline was taken from −500 ms to the stimulus onset, resulting in a time × frequency induced data set.

Both ERSs and ERDs were quantified according to the gradient of the change in synchronisation: linear regression was applied to the data collapsed across the frequency band pairs in the specified frequency range. This novel approach was adopted primarily because the rate of change over a fixed period following presentation of the stimuli was our primary interest. Although clearly proportional to the more commonly used methods of average amplitude deflection from baseline [94] or area under the curve [95], the rate of change of synchronisation (the gradient) should theoretically be less susceptible to outlying data points and potential confounds such as prior [75], [96] or subsequent amplitude fluctuations [84]. An attempt was made to apply a more conventional average amplitude change dependent variable to the ‘seen’ *vs*. ‘unseen’ correct contrast, but this resulted in a high level of outlier rejection according to Chauvenet's criterion.

**Figure 9:**
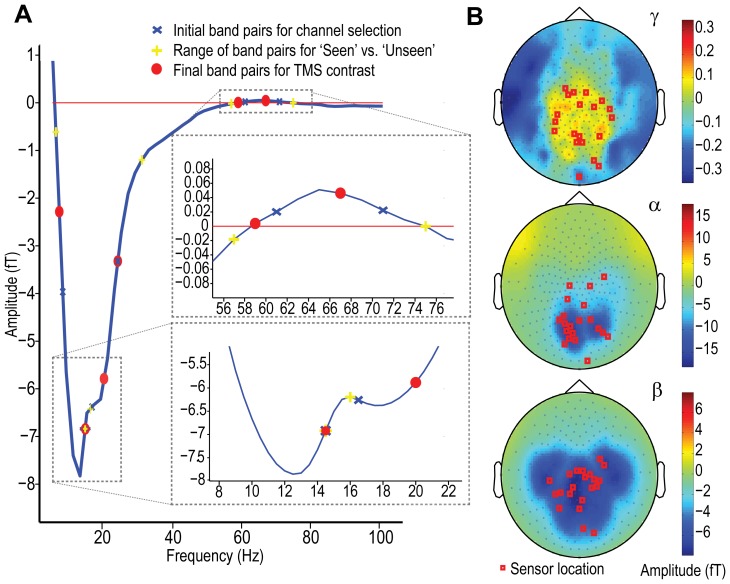
Experiment 4 – oscillatory results independent of TMS. A. Amplitude frequency distribution of combined (across ‘seen’ and ‘unseen’ correct) data sets under stimulus-present conditions, collapsed across time. Points highlighted are the initial frequency band pairings used for channel selection applied to the combined data set, the range of frequencies over which the ‘seen’ *vs*. ‘unseen’ correct contrast was applied, and the final set of band pairs upon which the dependent measures, applied to the TMS contrast, were based. B. Topographic distribution of synchrony levels and channels selected to be used in the ‘seen’ *vs*. ‘unseen’ correct contrast. Data used in the production of these plots was from the combined data set and averaged the oscillatory amplitude across the 0.1 to 0.4 second temporal epoch. Scales correspond to oscillatory amplitude in Tesla. Topographic plots are separated by the frequency bands over which mean amplitude was taken for channel section: i) γ ERS 61–71 Hz, ii) α ERD 8–14.5 Hz, and iii) β ERD 16.5–24 Hz.

**Figure 10:**
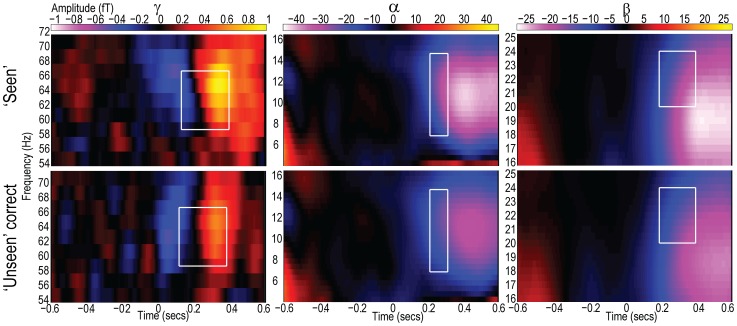
Experiment 4 – Time frequency responses independent of TMS. Time frequency induced responses to the presence of stimuli when ‘Seen’ and ‘Unseen’ yet direction is correctly discriminated, across TMS conditions. Depicted are the three frequencies examined (γ, α and β). The regions highlighted are those which express the greatest difference between ‘seen’ and ‘unseen’ correct conditions and were therefore applied to the subsequent contrasts across TMS conditions.

Channel selection based on the combined data sets required an initial set of parameters for each dependent measure (frequency and temporal ranges). To obtain these, full width half maximum (FWHM) criteria were applied to the group averaged amplitude spectrum of the combined induced responses during the 0.1 to 0.4 second period following the presentation of the stimuli. There were three dominant features of the amplitude spectrum: a low frequency desynchronisation, a reversal in amplitude separating α and β bands, and a synchronisation in the γ band (see Figure 9 and 10). The closest frequency to the midpoint between the start of the α ERD and its peak was at 8 Hz, and the midpoint between the peak and the α/β reversal was at 14.5 Hz. For β, the ERD midpoint between its peak and the reversal was at 16.5 Hz, and midway between the β peak and the end of the ERD was at 24 Hz. The γ ERS peaked at 65 Hz, indicating a FWHM range of 61 to 71 Hz. These parameters are illustrated in Figure 9A and the resulting channel sections are depicted in Figure 9B. Although the parameters were refined to express the maximal difference between ‘seen’ and ‘unseen’ correct trials, if the starting parameters used in channel selection are applied to the ‘seen’ *vs*. ‘unseen’ correct contrast, significant differences are expressed over all three frequency bands: α) 8–14.5 Hz, ‘seen’ *vs*. ‘unseen’ correct *t*_(21)_ = 6.29, *p* = 3.07e^−6^, *d* = 0.551, (N = 2 outliers excluded). β) 16.5–24 Hz, ‘seen’ *vs*. ‘unseen’ correct *t*_(23)_ =  3.78, *p* = 9.64e^−4^, *d* = 0.41. γ) 61–71 Hz. ‘seen’ *vs*. ‘unseen’ correct *t*_(21)_ = 2.62, *p* = 0.016, *d* = 0.70, (N = 1 outlier excluded).

The next stage in the development of the dependent variables was to search through and optimise the time and frequency parameters according to the ‘seen’ *vs*. ‘unseen’ correct contrast. T statistics over a range of potential parameter values were compared, and the parameters that resulted in the greatest difference between ‘seen’ and ‘unseen’ correct conditions were to be used to probe the TMS effects. Chauvenet's criterion was used to ensure that any difference was not the result of outlying data points. This involved exclusion of the parameter set that resulted in outlying data, rather than exclusion of outlier data points as with other applications of Chauvenet's criteria. The search for γ band parameters was constrained to where positive deflections in the combined data set were observed at 57 to 75 Hz, with a minimum difference between bands of 4 Hz incrementing at 0.5 Hz (see Figure 9A). In the temporal domain the search ranged from 0.05 to 0.4 seconds with a minimum step size of 0.067 seconds. In the α band the parameter search was applied to a range of 6 to 14.5 Hz and the temporal step size was 0.033 seconds. The same temporal constraints were applied to the β band but its potential frequency range was from 16 to 31 Hz.

Consistent with predictions, all dependent variables illustrated significant differences between the ‘seen’ and ‘unseen’ correct conditions, as illustrated in Figures 10. The resulting parameters and *t* statistics are as follows: γ ERS) 59 to 67 Hz band pairing was selected during a 0.128 to 0.395 second period following stimulus presentation (*t*_(23)_ = 2.83, *p* = 0.009, *d* = 0.74, Figure 9 and 10). α ERD) 7 to 14.5 Hz band pair and 0.218 to 0.318 second period were chosen (*t*_(23)_ = 6.04, *p* = 3.73e^−6^, *d* = 0.79, Figure 9 and 10). β ERD) 20 to 24 Hz band pair and 0.1946 to 0.398 second period was selected (*t*_(23)_ = 4.62, *p* = 1.20e^−4^,* d* = 0.52, Figure 9 and 10).

#### Application to TMS contrast

The data to which the time frequency and evoked analyses were applied consisted of all stimulus present trials in the data blocks, collapsed over pairs of behavioural blocks (see Figure 1C). Channel selection applied the same method as previously described, based upon peak deflection, but was applied here to each individual data block (two behavioural blocks), rather than the overall combined data set. The structure of the inferential analysis applied to the dependent measures, described above, was aligned with the behavioural analysis of Experiment 4. Repeated measures ANOVAs were applied to the Δ pre-TBS baseline data with 2 levels: site (cTBS *vs*. control) and time (the 4 post TBS blocks). Bayesian tests, as per other dependent measures, exploited the behavioural shift in conscious detection to specify the range of the hypothesis. As previously stated, this made use of the combined behavioural effect (reported as B_combined_). Additionally, it was possible to use the magnitude of the behavioural shift expressed by the same subjects to define the hypothesis. As with the pupillometry analysis, this involved using the standardised behavioural change observed in Experiment 4 only, resulting in a more specifically informed prior (reported as B_replication_). Therefore, for each dependent measure two Bayes factors are reported using combined (Experiment 1 and 4) and experiment-specific (Experiment 4 only) standardised shifts in PrC.

### 5.5. Experiment 4. MEG Results

To reiterate the hypothesis, the predicted cTBS upon the MEG measures is as follows: the application of cTBS might be accompanied by i) increased amplitude of later evoked responses that represent the potentiated transmission of conscious signals; ii) the increased synchronisation in the γ band, which would be similarly consistent with increased signal transmission, but may also be interpreted as reflecting increased segmentation of signals; iii & iv) Potentiation of the desynchronisation in either of the α (iii) or β (iv) bands indicating increased gating or active inhibition. It is worth noting that these last two predictions have a negative directionality due to their involving *de*synchronisations.

**i) ERF**

**Figure 11:**
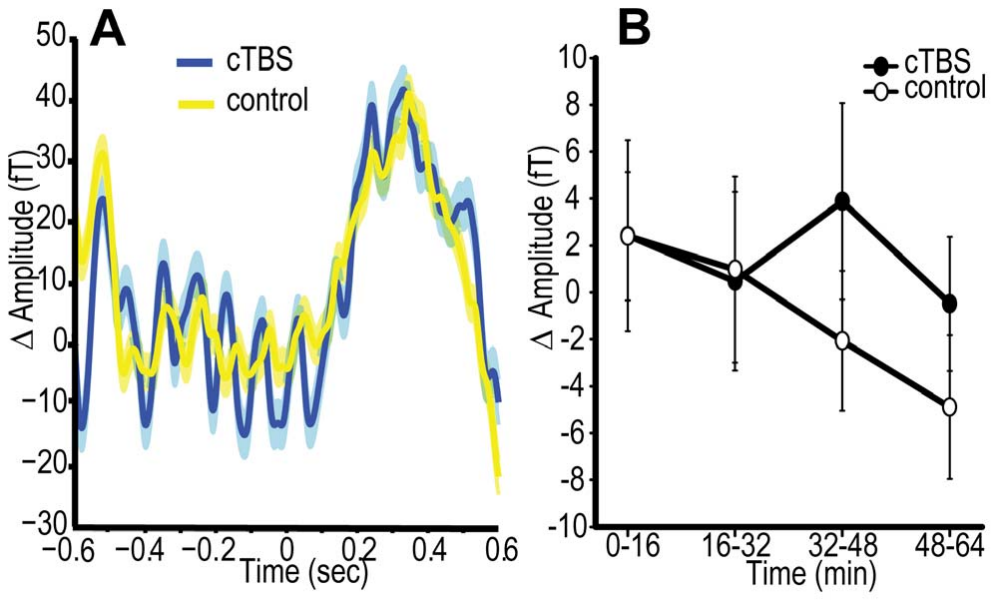
Experiment 4 – effects of cTBS on evoked responses. A. Group averaged evoked responses (ERF) following cTBS (blue) and control (yellow) stimulation, where shaded areas are one standard deviation across subjects. Plot derived from data averaged across post-TBS stimulus-present trials over occipital parietal clusters of channels. Δ refers to change from pre-stimulus baseline. B. Change from pre-TBS baseline in peak amplitude of evoked response following stimuli presentation for occipital/parietal channels. Active and control conditions are shown. cTBS *vs*. control *p* = 0.59, B_(cTBS>sham)_ = 0.57. Error bars are ±1 SEM.

Contrary to our hypothesis, we observed no discernible effect of the cTBS, compared to sham/iTBS control, upon evoked responses (see Figure 11). The ANOVA indicated that there was neither a significant main effect of site (cTBS *vs.* control, *F*_(1,22)_ = 0.31, *p* = 0.59, *d* = 0.14, N = 1 outlier excluded) nor a significant interaction between site × time post TBS (*F*_(3,66)_ = 0.63, *p* = 0.57). This was reflected by the Bayesian analysis, which supported the null hypothesis of an absence of positive effects, but not unequivocally (B_combined_ = 0.57, B_replication_ = 0.728). No time effect was observed in isolation (*F*_(3,66)_ = 0.83, *p* = 0.48).

**ii) γ ERS**

**Figure 12:**
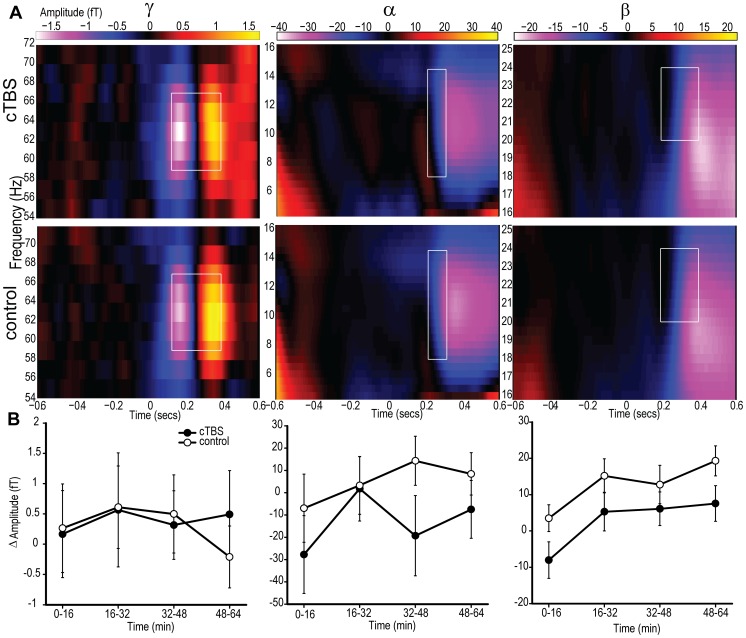
Experiment 4 – effects of cTBS on oscillatory responses. A. Time frequency induced responses to the presence of stimuli following cTBS and control stimulation. Data concatenated across post-TBS blocks. Highlighted regions indicate data used to derive dependent measures. B. Line plots illustrating change from pre-TBS baseine in the γ, α and β dependent measures under cTBS and control conditions. No statistically significant effects of the TMS were observed, but a trend for potentiated β ERD was observed which is consistent with previous research. γ cTBS *vs*. control *p* = 0.93, B_(γ cTBS>sham)_ = 0.44, α cTBS *vs*. control *p* = 0.28, B_(α cTBS<sham)_ = 0.96, β cTBS *vs*. control *p* = 0.12, B_(β cTBS<sham)_ = 1.85. Error bars are ±1 SEM.

The γ ERS hypothesis anticipated an increased induced γ band response following the application of the cTBS. No such effects were observed (see Figure 12, site cTBS *vs.* control *F*_(1,23)_ = 0.01, *p* = 0.93, *d* = 0.02, site × time interaction *F*_(3,69)_ = 0.25, *p* = 0.84). No time dependent changes were observed (*F*_(3,69)_ = 0.28, *p* = 0.77). The Bayesian analysis complemented the Neyman–Pearson statistics, supporting the null, but again not definitively (B_combined_ = 0.44, B_replication_ = 0.57).

**iii) α ERD**

According to the gating-by-inhibition hypothesis, the rate of the ERD might be expected to steepen in a negative direction following the application of cTBS relative to control stimulation. Although the direction of the mean shift was consistent with the prediction, it was not statistically significant (see Figure 12, site cTBS *vs.* control *F*_(1,22)_ = 1.21, *p* = 0.28, *d* = 0.27, N = 1 outlier excluded, site × time interaction *F*_(3,66)_ = 0.51, *p* = 0.63) and Bayesian analyses were inconclusive (B_combined_ = 0.959, B_replication_ = 1.189). No time dependent changes were observed (*F*_(3,66)_ = 0.86, *p* = 0.46).

**iv) β ERD**

As with the α ERD, increased negative desynchronisation might be expected to follow cTBS under the gating-by-inhibition hypothesis. A trend in this anticipated direction was observed, which did not interact significantly with time (see Figure 12, site cTBS *vs.* control *F*_(1,23)_ = 2.62, *p* = 0.12, *d* = 0.39, site × time interaction *F*_(3,66)_ = 0.14, p = 0.89). The Bayesian analysis also supported the existence of a potentiation of the ERD over the respective null, but not to the extent to provide strong support (B_combined_ = 1.85, B_replication_ = 2.19). Taken together with previous demonstrations of cTBS increasing β band response (e.g. [85], [86]), this data provides support, albeit weakly, for the gating-by-inhibition hypothesis.

The β ERD increased throughout the course of the experiment irrespective of the TMS interventions (see Figure 12, time effect *F*_(3,69)_ = 6.29, *p* = 0.001). Given that such low frequency oscillations are thought to index the inverse of levels of arousal [97], such a finding is consistent with increased fatigue throughout the course of the experiment, as previously suggested (see Sections 2.2 and 5.2).

## General Discussion

### Overview

Experiment 1 showed that reported awareness of stimuli was enhanced following occipital cTBS, whereas forced choice ‘unseen’ ability remained unaffected. This behavioural effect replicated successfully in Experiment 4, but with a smaller effect size. As cTBS is a neuronal suppressive protocol these effects ran counter to our *a priori* hypothesis. Subsequent experiments sought to explore the effect of the cTBS with a view to elucidating the neuronal basis of the behavioural change. Taken together, these findings suggest that consciousness arises, at least in part, from active inhibition in visual cortex, and that this inhibitory mechanism can be potentiated through inhibitory cortical stimulation (cTBS). In other words, cTBS suppressed neuronal noise, resulting in increased signal to noise ratio (SNR) and increased conscious detection.

### MRS and PT experiments

The inhibitory aspect of this explanation is perhaps most clearly evinced by the increase in the concentration of the principal inhibitory neurotransmitter GABA. One interpretation of our results is that the balance between excitation and inhibition is tipped in favour of inhibition by the application of cTBS, which in turn increases the contrast or gain between representations [24], [98]. GABAergic interneurons are thought to play an important role in this respect: if cortical responses are functionally understood as embodying an interplay between a model and a signal (or error signal), then the role played by the inhibitory/GABAergic system can be seen as critical by bringing the signal into alignment with the model [99]. A similar interpretation has been offered following the demonstration that microionophoretically (micro injection and measurement using ionic currents) applied GABA suppressed spontaneous discharges, but did not affect evoked response to auditory stimuli in rats [100]. The increase in observed GABA concentration following cTBS is therefore consistent with the gating-by-inhibition hypothesis, suggesting that an important factor in determining whether or not a representation is realised as conscious is active GABAergic inhibition.

A general suppression of neural activity caused by the cTBS is not sufficient in itself to explain the observed increase in conscious detection. Rather, we propose that the mechanisms that gate consciousness involve inhibition under normal conditions, and it was these selection mechanisms that were specifically facilitated by occipital cTBS. This may be why the application of cTBS resulted in the decreased detection of phosphenes and increased detection of normal external stimuli. Detection of phosphenes differs fundamentally from detection of external stimuli in that phosphenes are the result of direct pervasive stimulation of neurons in visual areas [35]. In contrast, conscious detection of external stimuli is the result of refined teleological mechanisms, which are likely to depend upon a fine balance between excitation and inhibition. Therefore, the increase in phosphene threshold is consistent with there being a general reduction in excitability and it is this inhibitory element – as part of the mechanism that produces conscious percepts – that benefits from the application of cTBS. The increase in phosphene threshold replicates the work of Franca et al.,[10], which supports the gating-by-inhibition hypothesis and detracts from the alternative hypothesis that cTBS might increase excitation and/or noise (stochastic resonance).

One aspect of the gating-by-inhibition hypothesis that is important to consider is why a moderate increased inhibition (due to cTBS) may lead to increased SNR through the suppression of noise, rather than reduced or maintained SNR via the suppression of signal. We speculate that this is due to the relative levels of activation involved in the signal compared to the noise. Noise presumably arises through a population of neurons being close to their threshold potential for discharge. Therefore, a small elevation in the level of inhibition will dramatically affect the likelihood of spontaneous discharge and levels of noise. By contrast, neuronal activations carrying signals result from the summation of post-synaptic currents *in addition* to spontaneous excitatory events. Therefore, the drive for signal related activity is higher than that involved in spontaneous noisy discharge and the effect of limited increment in inhibition will be expressed to a greater extent upon the prevalence of noise over signal.

### MEG experiment

The results of Experiment 4 demonstrated the classic evoked and oscillatory changes in responses to stimuli, where increased processing is associated with heightened field potentials [101], reduced low frequency oscillatory amplitude and increased high frequency amplitude [102]. We chose to quantify these changes in order to express neural correlates of consciousness, which in addition to being intrinsically informative could then be applied to the TMS contrast. This ‘functional localiser’ for the NCC involved comparing data when subjects reported being aware of stimuli to when they denied awareness yet were able to discriminate stimuli correctly. In one state, awareness is present, and in the other it is specifically lacking despite a closely related form of perception being present. Thus, the difference between these two states can be seen as specific to conscious processing.

Applying this contrast to the MEG data across TMS conditions revealed four reliable neural correlates of consciousness that are consistent with previous reports: the elevated late evoked response (e.g. [51]), the increased γ band ERS (e.g. [103]) and the increased α and β ERDs (e.g. [81], [104]). These inform the ongoing search for such correlates and were then used as probes to explore the effect of occipital cTBS.

If this task and contrast where to be developed in future work the experimenter might consider the use of discrimination tasks that include a greater number of potential incorrect responses. This would reduce the likelihood that some of the correct responses during the reportedly ‘unseen’ trials were the result of chance rather than illustrating residual unconscious capacity. Although this potentially weakens the ‘seen’ vs. ‘unseen’ correct contrast applied here (through the possibility that some of the ‘unseen’ correct trials contain no perceptual information) it does not negate the utility of the contrast altogether. This is because perception was clearly demonstrated in the majority of ‘unseen’ correct trials, as shown by the reliably above-chance discrimination performance.

Although there are inconsistencies in the reports made of the effects of rTMS upon neuro-magnetic/electric cortical responses (cf. [86], [105]) there are currently four independent studies which have highlighted changes in β frequency oscillations specifically as correlates of the application of cTBS to motor areas [85], [86], [87], [88]. These findings allow us to place greater confidence in the current demonstration of the increased β band response than would otherwise be the case given the statistical non-significance of our findings. Therefore, although the MEG data does not allow us to draw any strong conclusions, the trend for increased β ERD following cTBS weakly supports the gating-by-inhibition hypothesis

### Relation to previous research

The facilitation of conscious detection following cTBS here is by no means the first demonstration of improvements in cognitive capacities following repetitive TMS [77], [106], [107], [108], [109], [110], [111], [112], [113], [114]. Some of these investigations are less relevant to the interpretation of current experiments than others. In particular, some of these experiments employed high frequency protocols that are believed to increase activation of corresponding cortical representations [108], [111], [112], [114], unlike cTBS [9], [10]. For example, Tegenthoff et al., [111] used an excitatory 5 Hz TMS protocol to improve tactile discrimination when applied to somatosensory finger areas, and showed that this effect correlated with increased blood-oxygen-level-dependent activation in the corresponding region, as measured by functional magnetic resonance imaging. Other studies where improvements have been observed can be attributed to the artefactual effects of the TMS, such as auditory or inter-sensory facilitation [110]. Others still involve the improvement of clinical symptoms where cortical hyper-excitability is thought to be a cause of the affliction and, because of their clinical applicability, are some of the most promising avenues for research in the area. For instance, unilateral spatial neglect is thought to arise from hyper-excitability of the contralesional cortex [107], [115], [116]. Accordingly, increased inhibition following cTBS has been shown to relieve the symptoms of neglect [106], [107]. Similarly rTMS can help alleviate the symptoms of amblyopia [112] and speed verbal responses for aphasic patients following stroke [109]. In conjunction with our results, these studies suggest that the observed changes may possibly reflect part of a homeostatic response oriented towards the return of optimal conditions following the presumably high levels of enforced activity that occur during the application of cTBS.

Of greater relevance here is the study by Waterston and Pack [113] who reported that cTBS boosted visual sensitivity when applied at a similar intensity to the current study (they used 43% stimulator output, whereas we applied cTBS at 40.4%±5.2%SD in the original experiment and 41.9%±5.7%SD in the replication experiment). Waterston and Pack's conclusions are consistent with the data collected over the current experiments. In particular, they describe cTBS as being effective in improving ‘coarse’ perceptual judgements (using large angular displacement, low contrast gratings) and not ‘fine’ judgments (small angular displacement, high contrast gratings). It is notable that the detection task employed here more closely resembled the coarse task, consistent with their findings [113]. As here, they attribute the observed improvement in capacity to increased SNR through the suppression of noise. The mechanism they proposed also makes a clear prediction in relation to the levels of synchronised neuronal activity following the application of cTBS, which is potentially consistent with the β ERD effect observed in Experiment 4. Of particular note here is the work of Zohary et al. [117] who demonstrated that as the level of correlated neuronal activity is increased, a system's capacity to delineate signal from noise is reduced. Although not statistically significant, the most robust positive effect in the MEG data was the increment in the shift from the synchronised to a desynchronised/uncorrelated state, reflecting the predictions made by Waterston and Pack based on Zohary et al.

Although improvements following cTBS have been reported, suppression and disruption of capacities are perhaps more commonly reported (e.g. [10], [118]). By far the most relevant is the recent demonstration that cTBS applied to the occipital lobe decreased subjects' confidence and accuracy when performing a visual discrimination task [48]. How can the work of Rahnev et al.,[51] be reconciled with the data here?

The intensity at which Rahnev et al. applied the cTBS was higher than the intensity at which it was applied here (Rahnev et al., used 46.6%±18.9%SD, whereas Experiment 1 used 40.4%±5.2%SD and Experiment 4 used 41.9%±5.7%SD). This difference is likely because Rahnev et al. applied cTBS at 80% of phosphene threshold, which is commonly higher than motor threshold [21], [119]. Additionally, in the Rahnev et al. study, only the sessions where cTBS was applied to the occipital lobe commenced with a ‘hunting procedure’ in which relatively high intensity pulses were applied prior to the application of cTBS. Because such a procedure can reduce cortical excitability in isolation (as evidenced by increased phosphene threshold over the course of the phosphene threshold experiment in the sham cTBS condition, see Figure 4), this may be an additional source of neuronal suppression not applied here.

The main reason for the discrepancy between the results of Rahnev et al. and the current study may therefore be that they stimulated at a higher intensity than that applied here, resulting in greater suppression. If the gating-by-inhibition hypothesis is correct then it would assume that slight increases of inhibition can be conducive to optimal detection, particularly when stimuli are presented at peri-threshold levels against noise. However, when inhibition is increased to a greater extent, more pervasive suppression, including suppression of signals, can be expected with the consequence of reduced detection capacity (as per [48]). Additionally, as pointed out by Rahnev et al., it is likely that the effects of cTBS show a task-specificity. Here, unlike in their paradigm, the task involved presenting stimuli against a noisy background. If the gating-by-inhibition hypothesis is correct then the application of cTBS may only result in facilitation when successful task performance involves suppression of superfluous noisy representations. That is, the task here may simply be well suited to illustrating the facilitative aspect of cTBS.

There are two additional aspects of the study by Rahnev et al. that are worth highlighting. First, they used modelling based on SDT to delineate two possible explanations as to the possible effect of cTBS. From their data either the neuronal ‘signal’ was suppressed or neuronal ‘variance’ or noise was *increased*. The description of the effect here only directly contradicts the latter of these possibilities (increased noise). When Rahnev and colleagues applied the model to their data they found that suppressed signal better explained their behavioural shift. Indeed, the model indicated that reduced rather than increased variance/noise was expressed by their data, consistent with the interpretation offered here. Second, a key finding of the Rahnev et al. study was a demonstration of reduced short-range functional connectivity in the occipital lobe, following cTBS. Whilst this demonstration does not explain the discrepancy between the two studies, this finding is consistent with the gating-by-inhibition hypothesis, in that it is suggestive of cTBS causing a suppression of superfluous representations.

Although the effect of cTBS increasing conscious detection was replicated, two caveats warrant emphasis. First, some caution should be taken with respect to the reliability of the behavioural shift in conscious detection, as the replication of the behavioural effect was weaker than that of the original experiment, and the Bayes factor for the replication alone was inconclusive. Perhaps more importantly, the stimuli used here were presented amongst luminance noise and our proposed effect of cTBS can be interpreted in terms of suppression of superfluous noise. It is therefore possible that the increase in conscious detection may only be realised during tasks that embed task-relevant stimuli within noise, with the facilitation arising through suppression of that noise. That is, the effect may be stimulus-dependent [113]. Second, the TMS intensity applied in these experiments was relatively low. We predict that if the levels of TMS applied were to be raised, then so too would the levels of inhibition and a reversal of effects from facilitation to suppression might be expected. Accordingly, the ‘virtual lesion’ interpretation of rTMS (e.g. [120], [121]) may be inappropriate when applied to similar cTBS paradigms, and if an experimenter's intention is to cause clear suppression then we recommend that they consider using intensities greater than 80% of motor threshold and make adjustments for scalp-cortex distance (while also taking into account relevant safety considerations; see [19], [21], [26], [44]).

### Summary

These experiments explored a counterintuitive finding that a neuronally suppressive TMS protocol can enhance conscious detection of stimuli. Overall, our findings suggest that cTBS increased cortical inhibition, leading to increased signal to noise ratio through the suppression of noise. Gating by inhibition may therefore be pivotal for visual consciousness.
